# Safety and efficacy of pyronaridine–artesunate paediatric granules in the treatment of uncomplicated malaria in children: insights from randomized clinical trials and a real-world study

**DOI:** 10.1186/s12936-024-04885-3

**Published:** 2024-02-28

**Authors:** Michael Ramharter, Abdoulaye A. Djimde, Isabelle Borghini-Fuhrer, Robert Miller, Jangsik Shin, Adam Aspinall, Naomi Richardson, Martina Wibberg, Lawrence Fleckenstein, Sarah Arbe-Barnes, Stephan Duparc

**Affiliations:** 1 Department of Tropical Medicine, Bernhard Nocht Institute for Tropical Medicine, Hamburg, Germany; 2 Department of Medicine, University Medical Centre Hamburg-Eppendorf, Hamburg, Germany; 3 German Centre for Infection Research (DZIF), Partner Site Hamburg-Lübeck-Borstel, Hamburg, Germany; 4 Centre de Recherches Médicales de Lambaréné, Lambaréné, Gabon; 5 Malaria Research and Training Center (MRTC), Université des Sciences, des Techniques et des Technologies de Bamako (USTTB), Bamako, Mali; 6 Medicines for Malaria Venture, Route de Pré-Bois 20, 1215 Geneva 15, Switzerland; 7 Artemida Pharma, Stevenage, UK; 8 Shin Poong Pharm. Co. Ltd, Seoul, Korea; 9 Magenta Communications Ltd, Abingdon, UK; 10 DATAMAP GmbH, Freiburg, Germany; 11 Department of Pharmaceutical Sciences and Experimental Therapeutics, College of Pharmacy, University of Iowa, Iowa City, USA

**Keywords:** Malaria, *Plasmodium falciparum*, Pyronaridine–artesunate, Paediatric, Anti-malarial, Granule formulation

## Abstract

**Background:**

Children are particularly at risk of malaria. This analysis consolidates the clinical data for pyronaridine–artesunate (PA) paediatric granules in children from three randomized clinical trials and a real-world study (CANTAM).

**Methods:**

An integrated safety analysis of individual patient data from three randomized clinical trials included patients with microscopically-confirmed *Plasmodium falciparum*, body weight ≥ 5 kg to < 20 kg, who received at least one dose of study drug (paediatric safety population). PA was administered once daily for 3 days; two trials included the comparator artemether–lumefantrine (AL). PCR-adjusted day 28 adequate clinical and parasitological response (ACPR) was evaluated. Real-world PA granules safety and effectiveness was also considered.

**Results:**

In the integrated safety analysis, 63.9% (95% CI 60.2, 67.4; 426/667) of patients had adverse events following PA and 62.0% (95% CI 56.9, 66.9; 222/358) with AL. Vomiting was more common with PA (7.8% [95% CI 6.0, 10.1; 52/667]) than AL (3.4% [95% CI 1.9, 5.8; 12/358]), relative risk 2.3 (95% CI 1.3, 4.3; *P* = 0.004), occurring mainly following the first PA dose (6.7%, 45/667), without affecting re-dosing or adherence. Prolonged QT interval occurred less frequently with PA (3.1% [95% CI 2.1, 4.8; 21/667]) than AL (8.1% [95% CI 5.7, 11.4; 29/358]), relative risk 0.39 (95% CI 0.22, 0.67; *P* = 0.0007). In CANTAM, adverse events were reported for 17.7% (95% CI 16.3, 19.2; 460/2599) of patients, most commonly vomiting (5.4% [95% CI 4.6, 6.4; 141/2599]), mainly following the first dose, (4.5% [117/2599]), with all patients successfully re-dosed, and pyrexia (5.4% [95% CI 4.6, 6.3; 140/2599]). In the two comparative clinical trials, Day 28 ACPR in the per-protocol population for PA was 97.1% (95% CI 94.6, 98.6; 329/339) and 100% (95% CI 99.3, 100; 514/514) versus 98.8% (95% CI 95.7, 99.9; 165/167) and 98.4% (95% CI 95.5, 99.7; 188/191) for AL, respectively. In CANTAM, PA clinical effectiveness was 98.0% (95% CI 97.3, 98.5; 2273/2320).

**Conclusions:**

Anti-malarial treatment with PA paediatric granules administered once daily for 3 days was well tolerated in children and displayed good clinical efficacy in clinical trials, with effectiveness confirmed in a real-world study.

*Trial registration* Clinicaltrials.gov: SP-C-003-05: identifier NCT00331136; SP-C-007-07: identifier NCT0541385; SP-C-021-15: identifier NCT03201770. Pan African Clinical Trials Registry: SP-C-013-11: identifier PACTR201105000286876

**Supplementary Information:**

The online version contains supplementary material available at 10.1186/s12936-024-04885-3.

## Background

Malaria remains a formidable global health challenge particularly impacting children who lack sufficient immunity to the disease. In the case of *Plasmodium falciparum* malaria, delayed or ineffective treatment can cause progression to severe malaria, and potentially death. In 2022, around 76% of the estimated 608,000 deaths from malaria occurred in children under 5 years old, predominantly in sub-Saharan Africa [1]. Beyond the immediate threat to life, malaria in children can have enduring consequences, affecting cognitive development, impairing educational progress, and undermining overall health and well-being. In sub-Saharan Africa, school-aged children, 6 to 15 years old carry the highest burden of malaria, with around 200 million children at risk [2, 3]. Additionally, infections in children are a reservoir for further parasite transmission, hindering malaria control and elimination.

Effective treatment not only alleviates suffering and reduces mortality, but also contributes to breaking the cycle of transmission, thereby reducing the malaria burden and supporting the overall goal of malaria eradication. Addressing malaria in children necessitates an understanding of age-specific challenges. Children’s difficulty in taking tablets and the complexity of adjusting doses based on weight, especially when tablets must be crushed or divided [4], necessitates the development of effective and well tolerated paediatric formulations.

Pyronaridine–artesunate (PA) is an artemisinin-based combination therapy developed through a joint venture between the not-for-profit organization Medicines for Malaria Venture (Geneva, Switzerland) and the pharmaceutical company Shin Poong Pharm. Co. Ltd. (Seoul, Korea). The objective of the programme was to develop an affordable fixed-dose artemisinin-based combination therapy with high efficacy, good tolerability, dosed once-daily for 3 days and dosed irrespective of food intake. PA is the only artemisinin-based combination therapy granted a positive scientific opinion under the European Medicines Agency’s Article 58 procedure, with the adult tablets approved in 2012, and the paediatric granules in 2015. Both PA formulations are WHO Prequalified, included in the World Health Organization (WHO) Model List of Essential Medicines and Model List of Essential Medicines for Children and are in the WHO Guidelines for Malaria since November 2022 [5], as well as being approved by regulatory agencies in malaria endemic countries.

Across the clinical development programme, PA demonstrated good efficacy and tolerability against both *P. falciparum* in adults and children and *Plasmodium vivax* in adults [6–11]. A subsequent Phase 3b/4 study (WANECAM) confirmed the safety and efficacy of repeated PA treatment in adults and children assessed over 2 years [12, 13]. A local registration study in Kenya supported the use of PA in children 6 months to ≤ 12 years old [14]. Most recently, a large ‘real-world’, single arm, open-label investigational study (CANTAM) confirmed the acceptable safety, good tolerability, and high effectiveness of PA in the treatment of uncomplicated malaria in a more diverse study population, including patients with underlying liver function abnormalities, acute hepatitis, HIV, malnourished patients, children under 1 year of age, and women who were unknowingly pregnant [15].

The PA development programme was designed for concurrent investigation of both adult tablet and paediatric granule formulations [16]. As the development programme for PA was integrated for the two formulations, the findings for the studies including assessments of the PA paediatric granules were published across four papers, which included outcomes for both formulations [10–12, 15]. Hence, collated data for the granule formulation have not been published separately. It can, therefore, be difficult to obtain a clear overview of PA granules safety, efficacy and effectiveness in children across the different studies and settings. The aim of this analysis was to review the safety and efficacy of PA granules for the treatment of children with uncomplicated *P. falciparum* malaria across the core clinical development programme as well as findings from the large post-approval ‘real-world’ study.

## Methods

### Ethics statement

For each study, the protocol was approved by an independent ethics committee [10–12, 15]. All the studies were conducted in accordance with the Declaration of Helsinki (Tokyo 2004), Good Clinical Practice, and applicable regulations. Informed written or witnessed oral consent was obtained from all patients’ parents/guardians plus assent was required from children able to understand the study.

### Source studies

An overview of the studies included in this analysis is provided in Table 1 [10–12, 15]. All the Phase 2/3 trials that included paediatric *P. falciparum* malaria patients treated with PA paediatric granules (60/20 mg per sachet) were considered. These included a Phase 2 study conducted in Gabon (SP-C-003-05) in which one cohort of 15 patients received the granule formulation [10]; a multi-centre, randomized, open-label, comparative Phase 3 study (SP-C-007-07) of PA versus artemether–lumefantrine (AL) [11]; and a paediatric sub-study of a large, randomized, open-label, parallel 3-arm comparative trial of repeated anti-malarial treatment (SP-C-013-11; WANECAM) with PA versus AL or artesunate–amodiaquine (ASAQ) or dihydroartemisinin–piperaquine (DHA–PQP) versus AL or ASAQ [12]. Note that the aim of WANECAM was to assess the safety and efficacy of repeated treatment with PA, so patients received the same anti-malarial therapy for all their malaria episodes over a 2-year follow-up period. In the current analysis, only the first malaria episode was considered.

**Table 1:** Characteristics of the studies [10–12, 15]

Characteristic	SP-C-003-05	SP-C-007-07	SP-C-013-11 WANECAM	SP-C-021-15 CANTAM
Phase and design	Phase 2, open-label dose-escalation study	Phase 3, multi-centre, comparative, open-label, parallel-group, controlled clinical trial	Phase 3b/4, randomised, multicentre, open-label, longitudinal, controlled trial	Single-arm, open-label, cohort event monitoring study
Dates	Jun 2006 to Dec 2006	Nov 2007 to Sep 2008	Oct 2011 to Feb 2016	Jun 2017 to Apr 2019
Location and setting	One clinical unit in Gabon	Seven centres in Burkina Faso, Democratic Republic of Congo, Gabon, Côte d’Ivoire, Kenya, Mali, and the Philippines	Seven centres in Burkina Faso, Guinea, and Mali	Six centres in Cameroon, Democratic Republic of Congo, Gabon, Côte d’Ivoire, and Republic of Congo
Patients	Aged 2–14 years, body weight 10–40 kg	Aged ≤ 12 years, bodyweight ≥ 5 to < 25 kg	Adults and children aged 6 months and older, bodyweight ≥ 5 kg	Any age, bodyweight ≥ 5 kg
Treatments	Pyronaridine-artesunate 6:2 mg/kg, 9:3 mg/kg and 21:4 mg/kg tablets; 9:3 mg/kg granules	Pyronaridine–artesunate granules versus artemether–lumefantrine crushed tablets	Pyronaridine–artesunate (tablets or granules) or dihydroartemisinin–piperaquine (tablets or crushed tablets) versus either artesunate–amodiaquine (tablets or dissolved tablets) or artemether–lumefantrine (tablets or dispersible tablets)	Pyronaridine–artesunate (tablets and granules)
Primary outcomes	Tolerability, safety, and pharmacokinetics, PCR-adjusted ACPR at day 28	Day-28 PCR-adjusted ACPR > 90%, safety	2-year incidence rate of all malaria episodes; day 28 and 42 ACPR in uncomplicated malaria, safety	Hepatic event incidence, clinical effectiveness (see text for definitions)

*ACPR* adequate clinical and microbiological response, *PCR* polymerase chain reaction, *PP* per protocol, *ECG* electrocardiograph

Additionally, an analysis was conducted for patients administered PA granules in a single-arm, open-label cohort event monitoring study conducted across five Central and West African countries (SP-C-021-15; CANTAM) [15]. The main aim of CANTAM was to assess PA hepatic safety, tolerability, and effectiveness for the treatment of acute uncomplicated malaria, including patients with asymptomatic elevated baseline liver aminotransferases, under real-world conditions in Africa.

### Patients

Full details of the inclusion and exclusion criteria for the studies have been published [10–12, 15]. Briefly, for inclusion in the three randomized studies [10–12], eligible patients of either sex had microscopically-confirmed uncomplicated *P. falciparum* malaria (asexual parasite density 1000–200,000 μL^–1^ blood) and a history of fever, no signs of severe malaria, severe malnutrition, severe diarrhoea, or other significant disorders or febrile conditions. Patients were excluded in SP-C-003-05 and SP-C-007-07 if they had liver function test results more than 3 times above the upper limit of normal (> 3×ULN) or haemoglobin < 8 g/dL, and in WANECAM if they had an alanine aminotransferase (ALT) level > 2×ULN or haemoglobin < 7 g/dL. CANTAM was designed to reflect real-world clinical practice and patients were eligible for inclusion if they had malaria diagnosed by rapid diagnostic test or microscopy and no contraindications for PA treatment as per the approved Product Label (Summary of Product Characteristics) [15, 17].

For the PA granule formulation, patients in study SP-C-003-05 were aged > 2 to ≤ 14 years and body weight ≥ 10 to < 40 kg [10]. Patients in study SP-C-007-07 were aged ≤ 12 years old and body weight ≥ 5 kg to < 25 kg [11]. The prospectively planned analysis of patients receiving the PA granule formulation in the WANECAM study included patients ≥ 6 months old and body weight ≥ 5 to < 20 kg [12]. The post-hoc sub-analysis analysis of patients receiving PA granules in CANTAM included patients that received the granule formulation who weighed ≥ 5 to < 20 kg [15].

### Treatments

The PA granule formulation was manufactured and supplied by Shin Poong Pharm. Co., Ltd., Ansan, Korea. In study SP-C-003-05 the daily PA dose was 9:3 mg/kg (pyronaridine tetraphosphate:artesunate). In the other studies, PA granules were supplied in sachets (60:20 mg pyronaridine tetraphosphate:artesunate) and administered once daily for 3 days according to body weight. In SP-C-007-07 the dose was ≥ 5 to < 9 kg, one sachet (60:20 mg); ≥ 9 to < 17 kg, two sachets (120:40 mg); ≥ 17 to < 25 kg, three sachets (180:60 mg), giving a dose range of 6.7:2.2 to 13.3:4.4 mg/kg for each dose. In WANECAM and CANTAM the dose was ≥ 5 to < 8 kg, one sachet; ≥ 8 to < 15 kg, two sachets; ≥ 15 to < 20 kg, three sachets, giving a dose range 7.6:2.5 to 15.0:5.0 mg/kg for each dose. Granules were suspended in liquid and could be taken without regard to food intake.

In study SP-C-007-07, the comparator group included children randomized to AL tablets (20:120 mg) (Novartis SA, Basel, Switzerland) [11, 12]. AL was administered as crushed tablets suspended in water or milk and given with food, as per local guidelines twice daily for 3 days by body weight: ≥ 5 kg to < 15 kg, 1 tablet twice daily (40:240 mg/day) ≥ 15 to < 25 kg, 2 tablets twice daily (80:480 mg/day). In WANECAM, AL dispersible tablets were administered using the same dosing regimen as above in water with no requirements or restrictions on food intake [12]. ASAQ tablets (Sanofi, Paris, France) were dissolved in water and administered once daily for 3 days by body weight: ≥ 5 kg to < 9 kg, 1 tablet (25:67.5 mg/day); ≥ 9 to < 18 kg, 1 tablet (50:135 mg/day); and ≥ 18 to < 20 kg, 1 tablet (100:270 mg/day) with no restrictions on food intake [12].

For all treatments, vomiting within 30 min of the first dose resulted in repeated dosing. If the second dose was vomited, the patient was withdrawn from the study and rescue therapy administered. In SP-C-003-05, SP-C-007-07 and WANECAM all treatment doses were directly observed, whereas in CANTAM only the first treatment dose was supervised.

### Assessments

Full details of the assessments conducted in each study have been published [10–12, 15]. Briefly, following screening procedures, including a full medical history and physical examination, for SP-C-003-05, SP-C-007-07, and WANECAM, eligible patients received treatment for days 1 to 3 with follow up on days 7, 14, 21, 28, 35 and 42. In WANECAM, upon randomization patients received the same treatment for any subsequent episode of malaria occurring over 2 years. In CANTAM, patients were treated for days 1 to 3, with follow up on days 7 and 28.

Across all studies, Giemsa-stained thin and thick blood slides were prepared for parasite identification and quantification according to standard procedures [18]. Polymerase chain reaction (PCR) genotyping was used to differentiate between *P. falciparum* recrudescence and reinfection by comparing blood spot samples taken at baseline versus recurrence, using published methods [19].

Adverse events were assessed throughout the studies and categorized according to the MedDRA primary system organ class and preferred term. In study SP-C-003-05, clinical biochemistry and haematology was assessed at baseline, day 3 and 7 plus days 28 and 42 if clinically indicated, and electrocardiographs (ECGs) were performed at baseline, days 1, 2 and 28 plus days 7 and 14 if clinically indicated. In SP-C-007-07, venous blood samples for clinical biochemistry and haematology were taken at screening, days 3, 7, 28, and 42, urinalysis was performed at screening, and ECGs were done at screening, day 2, and if indicated at days 7, 14, and 28. In WANECAM, clinical biochemistry and haematology samples were collected pretreatment on days 0, 3, 7 and 28, and at other times if hepatic tests were abnormal or if deemed necessary by the investigator, ECGs were done on day 0 (pre-dose), day 2 (post-dose), and day 3 if clinically indicated. In CANTAM, baseline samples were collected for biochemistry, and post-baseline samples were only collected when clinically indicated. Haematology and ECGs were also only conducted when clinically indicated.

### Integrated safety evaluation

An integrated paediatric safety analysis of individual patient data from SP-C-003-05, SP-C-007-07 and WANECAM was conducted following a pre-determined statistical analysis plan using SAS®, Version 9.3 (SAS, Cary, NY) in a UNIX environment (Additional file 1). Safety outcomes were the incidence and severity of adverse events, and laboratory abnormalities. Safety outcomes were evaluated in the paediatric safety population, including all patients who weighed ≥ 5 kg and < 20 kg and who received at least one dose of the study drug. Patient age was calculated from the date of the screening visit. In the case of the 2-year follow-up longitudinal study WANECAM, integration of data with the other randomized clinical trials required that only the first treatment with PA or AL was considered and only patients that were directly randomized to PA or AL were included. A planned sub-group analysis considered the frequency of adverse events in patients with body weight ≥ 5 to < 8 kg, ≥ 8 to < 15 kg, and ≥ 15 to < 20 kg. Potential differences between PA and AL in adverse event incidence rates were analysed using Fisher’s exact test, with relative risk calculated post hoc. For key safety outcomes, 95% CIs were calculated (Wilson–Brown) (GraphPad Prism version 10.0.2, GraphPad Software, Inc., Boston, MA).

Separately, safety data on patients weighing ≥ 5 kg to < 20 kg who received PA granules were extracted from the CANTAM statistical outputs. Although the definition of the safety population for CANTAM and statistical treatment was consistent with the pooled analysis, because of the different study designs and settings, comparisons were not formally tested between the integrated safety analysis and CANTAM.

### Efficacy outcomes

The protocol-defined primary efficacy outcomes differed across the studies [10–12, 15]. Briefly, the intention-to-treat (ITT) population included all patients that received at least one dose of study drug. The per-protocol (PP) population included all patients in the ITT population who had a valid efficacy endpoint and no protocol violations that would impact efficacy outcomes.

Efficacy outcomes for SP-C-003-05, SP-C-007-07, and WANECAM were defined according to the WHO definition of adequate clinical and parasitological response (ACPR), i.e., clearance of asexual parasitaemia without recrudescence at the specified study day, irrespective of axillary temperature, without previous early treatment failure, late clinical failure, or late parasitological failure [18]. Primary efficacy outcomes were: SP-C-003-05, PCR-adjusted ACPR at day 28 in the PP population; SP-C-007-07, PCR-adjusted ACPR on day 28 > 90% in the PP population; and WANECAM, the 2-year incidence rate of all repeat malaria episodes (uncomplicated and complicated) irrespective of parasite species, and the PCR-adjusted and unadjusted ACPR at days 28 and 42. For the purposes of this analysis, PCR-adjusted ACPR at day 28 in the PP population for the first episode only was considered the key outcome of interest.

In CANTAM, the primary outcome was the hepatic event incidence, defined as the appearance of the clinical signs and symptoms of hepatotoxicity confirmed by a > 2× rise in ALT/AST versus baseline in patients with baseline ALT/AST > 2× the upper limit of normal (ULN) [15]. Clinical effectiveness was a secondary outcome, defined as the absence of microscopically-confirmed malaria without previous failure on day 28 in ITT and PP populations and is considered the key efficacy outcome of interest for this analysis [15].

## Results

### Safety population and drug exposure

#### Integrated safety analysis

**Fig. 1:**
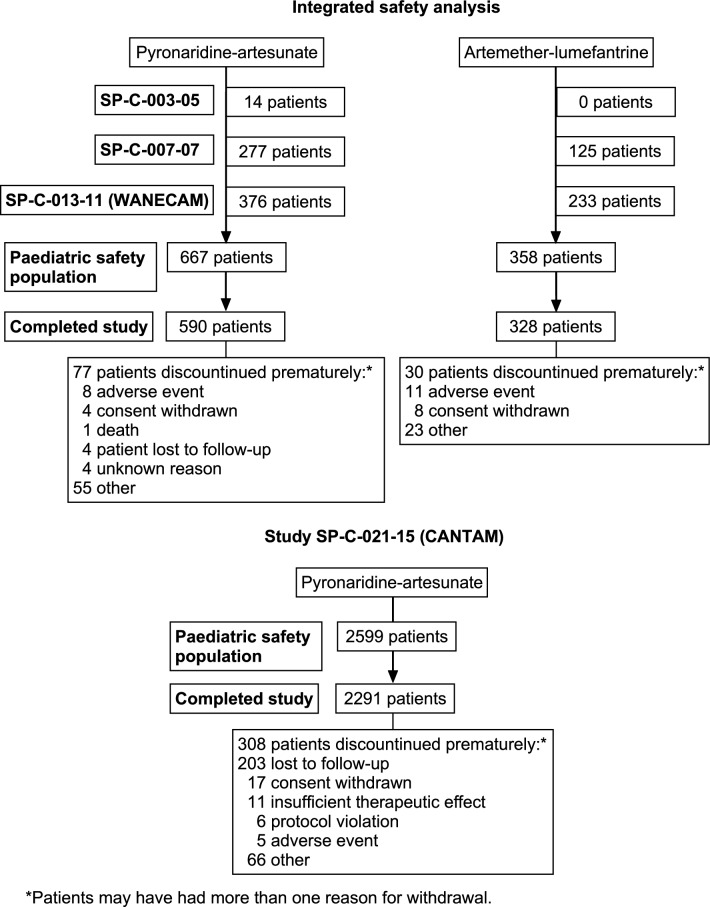
Disposition of the paediatric safety population

The paediatric safety population for the integrated safety analysis of SP-C-003-05, SP-C-007-07, and WANECAM included 667 patients who received PA granules, and 358 who received AL (Fig. 1). The baseline characteristics of the paediatric safety population are shown in Table 2. Most patients in both treatment groups were aged 3 to 5 years, with 3.0% of (20/667) patients in the PA group and 2.2% (8/358) in the AL group aged < 1 year. Mean total drug exposure over the three doses was 30.2 mg/kg for pyronaridine (standard deviation [SD] 5.8; range 8.2 to 63.8) and 10.1 mg/kg for artesunate (SD 1.9; range 2.7 to 21.3). Mean drug exposure for artemether was 12.1 mg/kg (SD 2.8; range 2.0 to 17.0) and 72.4 mg/kg for lumefantrine (SD 16.9; range 12.0 to 101.8).

**Table 2:** Baseline demographic and clinical characteristics for the integrated safety analysis and CANTAM

Characteristic	Integrated safety analysis		CANTAM PA (N = 2599)
	PA (N = 667)	AL (N = 358)	
Sex, n (%)			
Male	310 (46.5)	183 (51.1)	1283 (49.4)
Female	357 (53.5)	175 (48.9)	1316 (50.6)
Mean age, years (SD) [range]	4.1 (2.0) [0–10]	4.3 (2.0) [0–11]	3.5 (2.0) [0–17]
Age category, n (%)			
≤ 6 months	4 (0.6)	3 (0.8)	35 (1.3)
> 6 months to < 1 year	16 (2.4)	5 (1.4)	101 (3.9)
≥ 1 to < 3 years	131 (19.6)	55 (15.4)	769 (29.6)
≥ 3 to < 6 years	370 (55.5)	206 (57.5)	1258 (48.4)
≥ 6 years	146 (21.9)	89 (24.9)	436 (16.8)
Mean body weight, kg (SD) [range]	14.6 (3.1) [6.0–19.9]	14.9 (2.8) [7.2–19.9]	14.0 (3.3) [5.7–19.9]
Body weight category, n (%)			
≥ 5 to < 8 kg	7 (1.0)	3 (0.8)	70 (2.7)
≥ 8 to < 15 kg	319 (47.8)	165 (46.1)	1268 (48.8)
≥ 15 to < 20 kg	341 (51.1)	190 (53.1)	1261 (48.5)
Body mass index, kg/m^2^ (SD) [range]	14.7 (1.7) [6.0–24.5]	14.5 (1.6) [8.0–21.9]	14.9 (2.1) [7.9–32.0]

*PA* pyronaridine–artesunate, *AL* artemether–lumefantrine, *SD* standard deviation

In the integrated safety analysis, post-dose vomiting (within 30 min) with PA occurred most frequently on the first dose (6.7%, 45/667) and was less evident on the second (1.6%, 11/667) and third doses (0.9%, 6/667). Re-dosing with PA was usually successful with 1.1% (8/667) of patients vomiting. For AL, 2.2% (8/358) of patients vomited the first dose, 2.0% (7/358) the second, 1.1% (4/358) the third, 1.1% (4/358) the fourth, 0.3% (1/358) the fifth, and 0% (0/358) the sixth dose. Vomiting of AL following re-dosing occurred for 0.3% (1/358) of patients.

#### Real-world cohort event monitoring study

For CANTAM, the paediatric safety population comprised 2599 patients (Fig. 1). Most (48.4%, 1258/2599) were aged 3 to 5 years, with 5.2% (136/2599) aged < 1 year (Table 2). Most patients showed good adherence with 92.4% (2401/2599) of participants receiving three doses, 4.4% (114/2599) one or two doses and 3.2% (84/2599) four doses. Mean pyronaridine exposure per dose was 13.6 mg/kg (SD 4.4; range 3.1 to 42.1) and mean artesunate exposure was 4.5 mg/kg (SD 1.5; range 1.0 to 14.0). Post-dose vomiting of the first dose of PA occurred for 4.5% (117/2599) of patients, all of whom were successfully re-dosed, subsequent doses were unsupervised.

### Safety

#### Integrated safety analysis

In the integrated safety analysis, the frequency of adverse events of any cause for PA was 63.9% (95% CI 60.2, 67.4; 426/667), similar to AL with 62.0% (95% CI 56.9, 66.9; 222/358), relative risk 1.0 (95% CI 0.93, 1.1; *P* = 0.59) (Table 3). Study drug was discontinued because of an adverse event in 1.2% (95% CI 0.61, 2.3; 8/667) of patients with PA and 0.8% (95% CI 0.23, 2.4; 3/358) with AL.

**Table 3:** Adverse events in the integrated safety analysis and CANTAM

Adverse event	Integrated safety analysis						CANTAM PA (N = 2599)	
	PA (N = 667)		AL (N = 358)		Relative risk % (95% CI)	*P* value^a^		
	N	% (95% CI)	N	% (95% CI)			N	% (95% CI)
Any	426	63.9 (60.2, 67.4)	222	62.0 (56.9, 66.9)	1.0 (0.93, 1.1)	0.59	460	17.7 (16.3, 19.2)
Drug-related	200	30.0 (26.6, 33.6)	134	37.4 (32.6, 42.6)	0.80 (0.67, 0.96)	0.017	218	8.4 (7.4, 9.5)
Serious	8	1.2 (0.6, 2.3)	1	0.3 (0.01, 1.6)	4.3 (0.70, 26.4)	0.17	11	0.4 (0.2, 0.8)
Serious drug-related	4	0.6 (0.2, 1.5)	1	0.3 (0.01, 1.6)	2.1 (0.32, 14.3)	0.66	3	0.1 (0.03, 0.3)
Severe or life-threatening	11	1.6 (0.9, 2.9)	4	1.1 (0.4, 2.8)	1.5 (0.50, 4.4)	0.59	8	0.3 (0.2, 0.6)
Leading to death	1	0.1 (0.008, 0.8)	0	0 (0, 1.1)	NA	1.0	0	0 (0, 0.1)

NA: not applicable, cannot be calculated

^a^Pyronaridine–artesunate (PA) versus artemether–lumefantrine (AL)

**Fig. 2:**
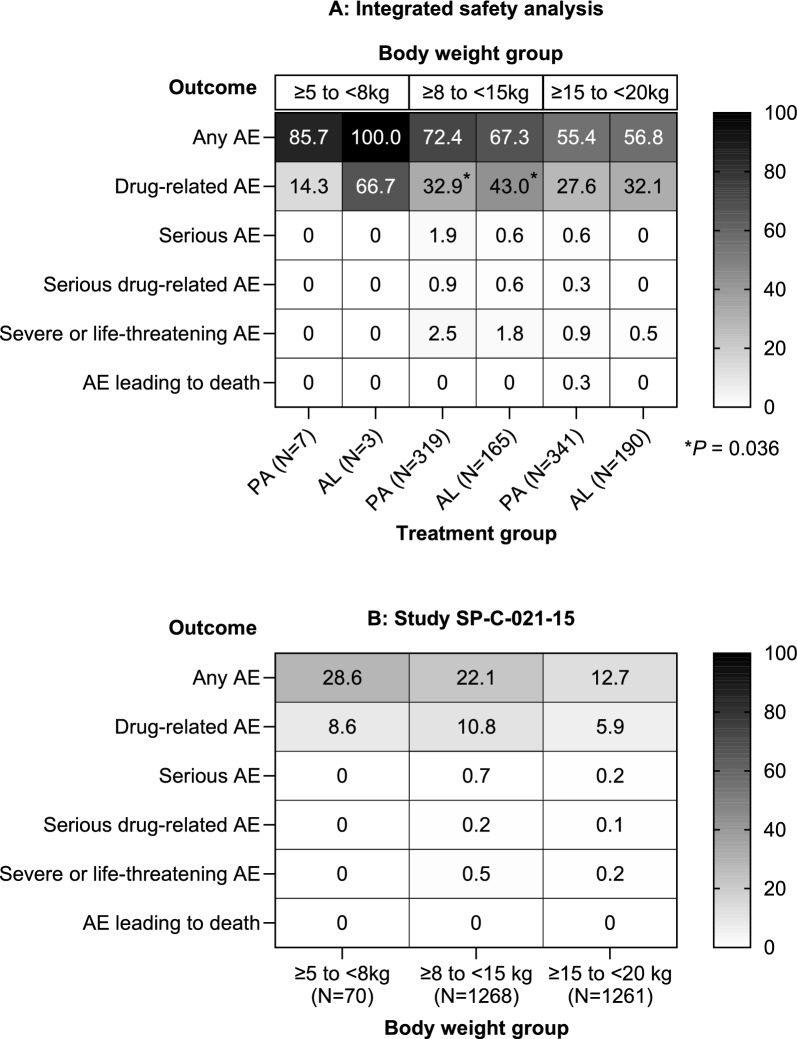
Overview of adverse events by body weight category. **A** Integrated safety analysis. **B** CANTAM (SP-C-021-15).  Data are the percentage of patients. *AE* adverse event, *AL* artemether–lumefantrine, *PA* pyronaridine–artesunate

For both PA and AL, adverse events of any cause were most common in patients with body weight ≥ 5 to < 8 kg followed by those weighing ≥ 8 to < 15 kg and ≥ 15 to < 20 kg (Fig. 2A). Drug-related adverse events in patients with body weight ≥ 8 to < 15 kg, were less frequent with PA (32.9% [95% CI 28.0, 38.2; 105/319]) compared with AL (43.0% [95% CI 35.7, 50.7; 71/165]), relative risk 0.76 (95% CI 0.61, 0.97; *P* = 0.036) (Fig. 2A). There were no significant differences between PA and AL in each body weight category for serious adverse events, serious drug-related adverse events, severe or life-threatening events or death (Fig. 2A).

The most common adverse events with PA were bronchitis (13.9%, 93/667) and vomiting (7.8%, 52/667), and for AL were bronchitis (14.8%, 53/358) and prolonged QT interval (8.1%, 29/358) (Table 4, Additional file 2). Mostly, the two drug therapies had a similar frequency of individual adverse events, except in the case of vomiting, prolonged QT interval and lymphocytosis (Additional file 2).

**Table 4:** Most common adverse events of any cause in the integrated safety analysis and CANTAM

Primary system class and preferred term	Integrated safety analysis					CANTAM PA (N = 2599)	
	PA (N = 667)		AL (N = 358)		*P* value^a^		
	N	% (95% CI)	N	% (95% CI)		N	% (95% CI)
Bronchitis	93	13.9 (11.5, 16.8)	53	14.8 (11.5, 18.9)	0.71	9	0.3 (0.2, 0.7)
Vomiting	52	7.8 (6.0, 10.1)	12	3.4 (1.9, 5.8)	0.004	141	5.4 (4.6, 6.4)
Cough	51	7.6 (5.9, 9.9)	24	6.7 (4.5, 9.8)	0.62	49	1.9 (1.4, 2.5)
Rhinitis	38	5.7 (4.2, 7.7)	27	7.5 (5.2, 10.8)	0.28	2	0.1 (0.01, 0.3)
Anaemia	35	5.2 (3.8, 7.2)	17	4.7 (3.0, 7.5)	0.77	20	0.8 (0.5, 1.2)
Upper RTI	34	5.1 (3.7, 7.0)	12	3.4 (1.9, 5.8)	0.21	0	(0, 0.1)
Platelet count increased	30	4.5 (3.2, 6.3)	17	4.7 (3.0, 7.5)	0.88	0	(0, 0.1)
Blood glucose decreased	27	4.0 (2.8, 5.8)	15	4.2 (2.6, 6.8)	1.00	0	(0, 0.1)
AST increased	26	3.9 (2.7, 5.7)	16	4.5 (2.8, 7.1)	0.74	0	(0, 0.1)
ECG QT prolonged	21	3.1 (2.1, 4.8)	29	8.1 (5.7, 11.4)	0.0007	0	(0, 0.1)
Pyrexia	20	3.0 (1.9, 4.6)	7	2.0 (1.0, 4.0)	0.41	140	5.4 (4.6, 6.3)
Influenza like illness	17	2.5 (1.6, 4.0)	4	1.1 (0.4, 2.8)	0.17	14	0.5 (0.3, 0.9)
Blood albumin decreased	17	2.5 (1.6, 4.0)	14	3.9 (2.3, 6.5)	0.25	0	(0, 0.1)
Neutropenia	15	2.2 (1.4, 3.7)	11	3.1 (1.7, 5.4)	0.41	0	(0, 0.1)
Abdominal pain	15	2.2 (1.4, 3.7)	7	2.0 (1.0, 4.0)	0.83	12	0.5 (0.3, 0.8)
ALT increased	15	2.2 (1.4, 3.7)	6	1.7 (0.8, 3.6)	0.65	0	(0, 0.1)
Blood potassium increased	15	2.2 (1.4, 3.7)	4	1.1 (0.4, 2.8)	0.23	0	(0, 0.1)
Haemoglobin decreased	14	2.1 (1.3, 3.5)	6	1.7 (0.8, 3.6)	0.81	1	0.04 (0.002, 0.2)
Nasopharyngitis	14	2.1 (1.3, 3.5)	3	0.8 (0.2, 2.4)	0.20	7	0.3 (0.1, 0.6)
Conjunctivitis	13	1.9 (1.1, 3.3)	5	1.4 (0.6, 3.2)	0.62	0	(0, 0.1)
Haematocrit decreased	13	1.9 (1.1, 3.3)	4	1.1 (0.4, 2.8)	0.44	0	(0, 0.1)
Headache	11	1.6 (0.9, 2.9)	2	0.6 (0.1, 2.0)	0.24	15	0.6 (0.4, 1.0)
Monocytosis	10	1.5 (0.8, 2.7)	10	2.8 (1.5, 5.1)	0.16	0	(0, 0.1)
Helminthic infection	9	1.3 (0.7, 2.5)	2	0.6 (0.1, 2.0)	0.35	0	(0, 0.1)
WBC count increased	9	1.3 (0.7, 2.5)	2	0.6 (0.1, 2.0)	0.35	0	(0, 0.1)
Decreased appetite	8	1.2 (0.6, 2.3)	1	0.3 (0.14, 1.6)	0.17	13	0.5 (0.3, 0.9)
Infection parasitic	7	1.0 (0.5, 2.2)	1	0.3 (0.14, 1.6)	0.27	0	(0, 0.1)
Splenomegaly	6	0.9 (0.4, 1.9)	4	1.1 (0.4, 2.8)	0.75	2	0.1 (0.01, 0.3)
Diarrhoea	6	0.9 (0.4, 1.9)	4	1.1 (0.4, 2.8)	0.75	37	1.4 (1.0, 2.0)
Blood creatinine decreased	5	0.7 (0.3, 1.7)	6	1.7 (0.8, 3.6)	0.21	0	(0, 0.1)
Varicella	4	0.6 (0.2, 1.5)	4	1.1 (0.4, 2.8)	0.46	1	0.04 (0.002, 0.1)
Pneumonia	3	0.4 (0.1, 1.3)	5	1.4 (0.6, 3.2)	0.14	2	0.1 (0.01, 0.3)
Enterocolitis	1	0.1 (0.008, 0.8)	4	1.1 (0.4, 2.8)	0.053	0	(0, 0.1)

Adverse events occurring in at least 1% of patients in any treatment group are included. The full list of adverse events is available in Additional file 2

*ALT* alanine aminotransferase, *AST* aspartate aminotransferase, *ECG* electrocardiograph, *NA* not applicable, *RTI* respiratory tract infection, *WBC* white blood cell

^a^Pyronaridine–artesunate (PA) versus artemether–lumefantrine (AL)

Vomiting was more common with PA (7.8% [95% CI 6.0, 10.1; 52/667]) than with AL (3.4% [95% CI 1.9, 5.8; 12/358]), relative risk 2.3 (95% CI 1.3, 4.3; *P* = 0.004). With PA, vomiting was mild for 7.3% (49/667) of patients and moderate for 0.4% (3/667), with no instances of severe vomiting. For AL, vomiting was mild for 2.8% (10/358), moderate for 0.3% (1/358), and severe for 0.3% (1/358). When analysed by body weight, vomiting was most common in patients with body weight < 8 kg for PA and AL, but numbers were small (Table 5). Vomiting was more common with PA in patients weighing ≥ 8 to < 15 kg (12.5% [95% CI 9.3, 16.6; 40/319]) compared with AL (4.2% [95% CI 2.1, 8.5; 7/165]), relative risk 3.0 (95% CI 1.4, 6.4; *P* = 0.003. There was no significant difference in the vomiting frequency between PA and AL within the < 8 kg (*P* = 1.00) or the ≥ 15 to < 20 kg (*P* = 0.78) body weight sub-groups (Table 5).

**Table 5:** Incidence of vomiting and prolonged QT interval by body weight in the integrated safety analysis and CANTAM

Adverse event by body weight	Integrated safety analysis						CANTAM^b^ PA (N = 2599)	
	PA (N = 667)		AL (N = 358)		Relative risk % (95% CI)	*P* value^a^		
	n/N	% (95% CI)	n/N	% (95% CI)			n/N	% (95% CI)
Vomiting								
≥ 5 to < 8 kg	2/7	28.6 (5.1, 64.1)	1/3	33.3 (1.7, 88.2)	0.86 (0.16, 5.7)	1.00	4/70	5.7 (2.2, 13.8)
≥ 8 to < 15 kg	40/319	12.5 (9.3, 16.6)	7/165	4.2 (2.1, 8.5)	3.0 (1.4, 6.4)	0.003	100/1268	7.9 (6.5, 9.5)
≥ 15 to < 20 kg	10/341	2.9 (1.6, 5.3)	4/190	2.1 (0.8, 5.3)	1.4 (0.47, 4.2)	0.78	37/1261	2.9 (2.1, 4.0)
Prolonged QT interval								
≥ 5 to < 8 kg	0/7	0 (0, 35.4)	1/3	33.3 (1.7, 88.2)	0 (0, 1.5)	0.30	0/70	0 (0, 5.2)
≥ 8 to < 15 kg	13/319	4.1 (2.4, 6.8)	18/165	10.9 (7.0, 16.6)	0.37 (0.19, 0.73)	0.006	0/1268	0 (0, 0.30)
≥ 15 to < 20 kg	8/341	2.3 (1.2, 4.6)	10/190	5.3 (2.9, 9.4)	0.45 (0.18, 1.1)	0.084	0/1261	0 (0, 0.30)

^a^Pyronaridine–artesunate (PA) versus artemether–lumefantrine (AL)

^b^Note that electrocardiographs were only to be performed if clinically indicated in the CANTAM study whereas they were part of the clinical protocol for the studies included in the integrated safety analysis

Prolonged QT interval (> 450 ms) occurred less frequently with PA (3.1% [95% CI 2.1, 4.8; 21/667]) than with AL (8.1% [95% CI 5.7, 11.4; 29/358]), relative risk 0.39 (95% CI 0.23, 0.67; *P* = 0.0007) (Table 4). When analysed by body weight, the frequency of prolonged QT interval was significantly lower with PA (4.1% [95% CI 2.4, 6.8; 13/319]) than AL (10.9% [95% CI 7.0, 16.6; 18/165]) for patients weighing ≥ 8 to < 15 kg, relative risk 0.37 (95% CI 0.19, 0.73; *P* = 0.006) (Table 5). There was no significant difference in the frequency of prolonged QT interval between PA and AL within the < 8 kg (*P* = 0.30) or the ≥ 15 to < 20 kg (*P* = 0.08) body weight sub-groups (Table 5).

Lymphocytosis was not reported as an adverse event for patients receiving PA (0% [95% CI 0, 0.6; 0/667), but was noted for 0.8% (95% CI 0.2, 2.4; 3/358) of patients receiving AL, relative risk 0.0 (95% CI 0, 0.6; *P* = 0.04). However, this adverse event was uncommon and the overall haematology findings for the two artemisinin-based combination therapies were similar (see below).

In patients who experienced an adverse event, most were of mild-to-moderate severity (grade 1 or 2) in both the PA (97.7% [95% CI 95.7, 98.7; 416/426]) and AL groups (98.2%, [95% CI 95.5, 99.3; 218/222]) (Additional file 3). In the PA group 7/667 (1.0% [95% CI 0.5, 2.2]) patients had nine grade 3 adverse events, and 3/667 (0.4% [95% CI 0.1, 1.3]) patients had four grade 4 adverse events (Additional file 3). With AL, 4/358 (1.1% [95% CI 0.4, 2.8]) patients had six grade 3 events and there were no grade 4 (0% [95% CI 0, 1.1]) adverse events (Additional file 3).

Drug-related adverse events were less frequent with PA (30.0% [95% CI 26.6, 33.6; 200/667]) than AL (37.4% [95% CI 32.6, 42.6; 134/358]), relative risk 0.8 (95% CI 0.67, 0.96; *P* = 0.017) (Table 3; Additional file 4). The most common drug-related adverse event with PA was vomiting (4.8% [95% CI 3.4, 6.7; 32/667]), though the incidence was not significantly different to with AL (3.1% [95% CI 1.7, 5.4; 11/358]), relative risk 1.6 (95% CI 0.81, 3.0; *P* = 0.25) (Table 6, Additional file 4). There was a significantly lower incidence of prolonged QT interval with PA (3.0% [95% CI 1.9, 4.6; 20/667]) versus AL (7.8% [95% CI 5.5, 11.1; 28/358]), relative risk 0.38 (95% CI 0.22, 0.67; *P* = 0.0009). Bronchitis was also less common with PA (1.0% [95% CI 0.5, 2.2; 7/667]) versus AL (3.1% [95% CI 1.7, 5.4; 11/358]), relative risk 0.34 (95% CI 0.14, 0.85; *P* = 0.024), as was lymphocytosis (0% [95% CI 0, 0.6; 0/667) versus 0.8% [95% CI 0.23, 2.4; 3/358], respectively, relative risk 0.0 (95% CI 0, 0.7; *P* = 0.04).

**Table 6:** Most common drug-related adverse events in the integrated safety analysis and CANTAM

Primary system class and preferred term	Integrated safety analysis					CANTAM PA (N = 2599)	
	PA (N = 667)		AL (N = 358)		*P* value^a^		
	N	% (95% CI)	N	% (95% CI)		N	% (95% CI)
Vomiting	32	4.8 (3.4, 6.7)	11	3.1 (1.7, 5.4)	0.25	109	4.2 (3.5, 5.0)
Blood glucose decreased	25	3.7 (2.6, 5.5)	12	3.4 (1.9, 5.8)	0.86	0	0 (0, 0.1)
Platelet count increased	25	3.7 (2.6, 5.5)	13	3.6 (2.1, 6.1)	1.0	0	0 (0, 0.1)
AST increased	21	3.1 (2.1, 4.8)	15	4.2 (2.6, 6.8)	0.38	0	0 (0, 0.1)
ECG QT prolonged	20	3.0 (1.9, 4.6)	28	7.8 (5.5, 11.1)	0.0009	0	0 (0, 0.1)
Anaemia	16	2.4 (1.5, 3.9)	13	3.6 (2.1, 6.1)	0.32	4	0.2 (0.1, 0.4)
Blood albumin decreased	15	2.2 (1.4, 3.7)	14	3.9 (2.3, 6.5)	0.17	0	0 (0, 0.1)
Neutropenia	15	2.2 (1.4, 3.7)	8	2.2 (1.1, 4.3)	1.0	0	0 (0, 0.1)
ALT increased	14	2.1 (1.3, 3.5)	6	1.7 (0.8, 3.6)	0.81	0	0 (0, 0.1)
Blood potassium increased	14	2.1 (1.3, 3.5)	4	1.1 (0.4, 2.8)	0.32	0	0 (0, 0.1)
Haemoglobin decreased	13	1.9 (1.1, 3.3)	5	1.4 (0.6, 3.2)	0.62	1	0.04 (0.002, 0.2)
Haematocrit decreased	12	1.8 (1.0, 3.1)	4	1.1 (0.4, 2.8)	0.60	0	0 (0, 0.1)
Upper RTI	11	1.6 (0.9, 2.9)	4	1.1 (0.4, 2.8)	0.59	0	0 (0, 0.1)
Monocytosis	8	1.2 (0.6, 2.3)	9	2.5 (1.3, 4.7)	0.13	0	0 (0, 0.1)
WBC increased	8	1.2 (0.6, 2.3)	1	0.3 (0.014, 1.6)	0.17	0	0 (0, 0.1)
Bronchitis	7	1.0 (0.5, 2.2)	11	3.1 (1.7, 5.4)	0.02	0	0 (0, 0.1)
Abdominal pain	6	0.9 (0.4, 1.9)	4	1.1 (0.4, 2.8)	0.75	7	0.3 (0.1, 0.6)
Blood creatinine decreased	5	0.7 (0.3, 1.7)	6	1.7 (0.8, 3.6)	0.21	0	0 (0, 0.1)
Rhinitis	3	0.4 (0.1, 1.3)	4	1.1 (0.4, 2.8)	0.25	0	0 (0, 0.1)
Pyrexia	1	0.1 (0.008, 0.8)	0	0 (0, 1.1)	1.0	25	1.0 (0.7, 1.4)

Drug-related adverse events occurring in at least 1% of patients in any treatment group are included. The full list of drug-related adverse events is available in Additional file 4

*ALT* alanine aminotransferase, *AST* aspartate aminotransferase, *ECG* electrocardiograph, *NA* not applicable, *RTI* respiratory tract infection, *WBC* white blood cell

^a^Pyronaridine–artesunate (PA) versus artemether–lumefantrine (AL)

Serious adverse events occurred in 1.2% (95% CI 0.6, 2.3; 8/667) of patients in the PA group and 0.3% (95% CI 0.01. 1.6; 1/358) in the AL group (Table 3), all of which occurred in patients with body weight ≥ 8 kg (Table 7). Serious adverse events considered drug related occurred in four patients in the PA group (five events) and in one patient (one event) in the AL group (Tables 3, 7). All other serious adverse events resolved. There was one death from multi-organ failure following a road traffic accident which was not considered related to study medication.

**Table 7:** Serious adverse events by body weight category in the integrated safety analysis and CANTAM

Body weight category Preferred term	Integrated safety analysis					CANTAM PA (N = 2599)	
	PA		AL		*P* value^a^		
	N	% (95% CI)	N	% (95% CI)			
Body weight ≥ 8 to < 15 kg	319		165			1268	
At least one adverse event	6	1.9 (0.9, 4.0)	1	0.6 (0.03, 3.4)	0.43	9	0.7 (0.4, 1.3)
Drug-induced liver injury	1^b^	0.3 (0.02, 1.8)	0	0 (0, 2.3)	1.00	0	0 (0, 0.3)
Malaria	3	0.9 (0.3, 2.7)	0	0 (0, 2.3)	0.55	3	0.2 (0.06, 0.7)
Transaminases increased	2^b^	0.6 (0.1, 2.6)	0	0 (0, 2.3)	0.55	0	0 (0, 0.3)
Toxic epidermal necrolysis	0	0 (0, 1.2)	1^b^	0.6 (0.03, 3.4)	0.34	0	0 (0, 0.3)
Anaemia	0	0 (0, 1.2)	0	0 (0, 2.3)	NA	5^b^	0.4 (0.2, 0.9)
Sepsis	0	0 (0, 1.2)	0	0 (0, 2.3)	NA	1	0.1 (0.004, 0.4)
Stevens–Johnson syndrome	0	0 (0, 1.2)	0	0 (0, 2.3)	NA	1	0.1 (0.004, 0.4)
Body weight ≥ 15 to < 20 kg	341		190			1261	
At least one adverse event	2	0.6 (0.1, 2.1)	0	0 (0, 2.0)	0.54	2	0.2 (0.03, 0.6)
Anaemia	1^b^	0.3 (0.02, 1.6)	0	0 (0, 2.0)	1.00	1	0.1 (0.004, 0.4)
Multi-organ failure	1	0.3 (0.02, 1.6)	0	0 (0, 2.0)	1.00	0	0 (0, 0.3)
Malaria	1^b^	0.3 (0.02, 1.6)	0	0 (0, 2.0)	1.00	1	0.1 (0.004, 0.4)
Epistaxis	0	0 (0, 1.1)	0	0 (0, 2.0)	NA	1^b^	0.1 (0.004, 0.4)

There were no serious adverse events in patients with body weight ≥ 5 to < 8 kg

*NA* not applicable

^a^Pyronaridine–artesunate (PA) versus artemether–lumefantrine (AL)

^b^In the integrated safety analysis there were five serious adverse events considered drug related with PA and one with AL. In CANTAM, two cases of anaemia and one of epistaxis were considered related to drug treatment

Post-baseline haematology and clinical biochemistry findings were comparable between PA and AL (Additional file 5). The proportion of patients with a decline in haemoglobin > 20 g/L from baseline at day 3 was 7.5% (95% CI 5.7, 9.8; 48/637) with PA and 10.5% (95% CI 7.7, 14.2; 36/343) with AL, and at day 7 was 5.8% (95% CI 4.3, 7.9; 37/634) and 7.6% (95% CI 5.3, 10.9; 26/341), respectively. By day 28, haemoglobin had recovered to at least baseline levels in 74.4% (95% CI 70.8, 77.7; 459/617) of patients in the PA group and 72.2% (95% CI 67.0, 76.8; 231/320) in the AL group.

**Fig. 3:**
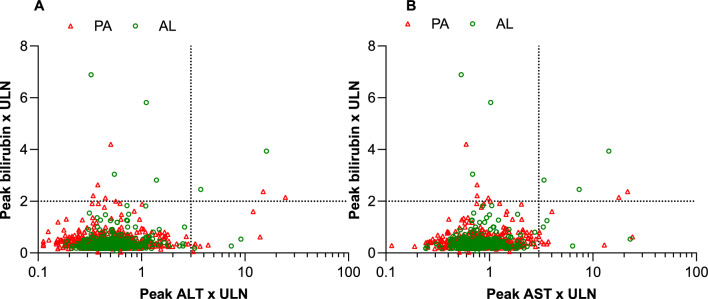
Integrated safety analysis peak bilirubin versus **A** peak ALT and **B** peak AST. Values are from day 3 until day 28 inclusive. *ALT* alanine aminotransferase, *AL* artemether–lumefantrine, *AST* aspartate aminotransferase, *PA* pyronaridine–artesunate, *ULN* upper limit of normal

There was no difference in the frequency of elevated ALT, AST or bilirubin between PA and AL (Table 8). Potential Hy’s Law cases occurred in 0.3% (95% CI 0.1, 1.1; 2/650) of evaluable patients in the PA group and 0.9% (95% CI 0.2, 2.5; 3/349) in the AL group, relative risk 0.36 (95% CI 0.072, 1.8) (*P* = 0.35) (Table 8, Fig. 3). In the PA group, one case was in a 3-year-old (11 kg) who had mild/moderate ALT, alkaline phosphatase (AP) and bilirubin elevation at baseline which had increased at day 7 to ALT 15×ULN, total bilirubin 2.4×ULN and AP 1.4×ULN, with no clinical signs and resolution by day 28; there was also exposure to traditional medicine at day 3. The second case was in a 2-year-old (10.2 kg), with day 7 ALT 24×ULN, total bilirubin 2.1×ULN and AP 1.8×ULN, with no clinical signs and normalization by day 28; the patient also received paracetamol and metamizole. In this case, repeated exposure to PA therapy 118 days later did not provoke a notable increase in liver enzymes [12].

**Table 8:** Hepatic safety measures in the integrated safety analysis

	PA (N = 667)		AL (N = 358)		*P* value^a^
	N	% (95% CI)	N	% (95% CI)	
ALT	650		350		
≤ 1×ULN	622	95.7 (93.8, 97.0)	337	96.3 (93.7, 97.8)	0.74
> 1.5×ULN to ≤ 3×ULN	19	2.9 (1.9, 4.5)	8	2.3 (1.2, 4.4)	0.68
> 3×ULN to ≤ 5×ULN	4	0.6 (0.2, 1.6)	2	0.6 (0.1, 2.1)	1.00
> 5×ULN to ≤ 10×ULN	1	0.2 (0.008, 0.9)	2	0.6 (0.1, 2.1)	0.28
> 10×ULN	4	0.6 (0.2, 1.6)	1	0.3 (0.015, 1.6)	0.66
AST	650		350		
≤ 1×ULN	577	88.8 (86.1, 91.0)	319	91.1 (87.7. 93.7)	0.28
> 1.5×ULN to ≤ 3×ULN	61	9.4 (7.4, 11.9)	24	6.9 (4.7, 10.0)	0.19
> 3×ULN to ≤ 5×ULN	7	1.1 (0.5, 2.2)	3	0.9 (0.2, 2.5)	1.00
> 5×ULN to ≤ 10×ULN	0	0 (0, 0.6)	2	0.6 (0.1, 2.1)	0.12
> 10×ULN	5	0.8 (0.3, 1.8)	2	0.6 (0.1, 2.1)	1.00
Total bilirubin	650		349		
≤ 1.5×ULN	636	97.8 (96.4, 98.7)	340	97.4 (95.2, 98.6)	0.66
> 1.5×ULN to ≤ 2×ULN	8	1.2 (0.6, 2.4)	3	0.9 (0.2, 2.5)	0.76
> 2×ULN to ≤ 3×ULN	5	0.8 (0.3, 1.8)	2	0.6 (0.1, 2.1)	1.00
> 3×ULN	1	0.2 (0.008. 0.9)	4	1.1 (0.4, 2.9)	0.053
Potential Hy’s Law^b^	2	0.3 (0.1, 1.1)	3	0.9 (0.2, 2.5)	0.35

*ALT* alanine aminotransferase, *AST* aspartate aminotransferase, *ULN* upper limit of normal

^a^Pyronaridine phosphate–artesunate (PA) versus artemether–lumefantrine (AL)

^b^Alanine aminotransferase or aspartate aminotransferase > 3×ULN and total bilirubin > 2×ULN

#### Real-world cohort event monitoring study

In CANTAM, the frequency of adverse events of any cause with PA was at 17.7% (95% CI 16.3, 19.2; 460/2599) (Table 3). This is lower than for the integrated safety analysis partly because adverse events associated with investigations (blood chemistry, urinalysis, ECG) were only conducted if clinically indicated (Table 4). Adverse events with PA were most common in the ≥ 5 to < 8 kg body weight group, occurring in 28.6% (95% CI 19.3, 40.1; 20/70) of patients, compared with 22.1% (95% CI 19.9, 24.4; 280/1268) in the ≥ 8 to < 15 kg and 12.7% (95% CI 11.0, 14.6; 160/1261) in the ≥ 15 to < 20 kg sub-groups (Fig. 2B).

The most common adverse events with PA were vomiting (5.4% [95% CI 4.6, 6.4; 141/2599]) and pyrexia (5.4% [95% CI 4.6, 6.3; 140/2599]) (Table 4). Vomiting following PA occurred in 5.7% (95% CI 2.2, 13.8; 4/70) of patients with body weight < 8 kg, 7.9% (95% CI 6.5, 9.5; 100/1268) of those ≥ 8 to < 15 kg and 2.9% (95% CI 2.1, 4.0; 37/1261) of those ≥ 15 to < 20 kg (Table 5). Drug-related adverse events occurred in 8.4% (7.4, 9.5; 218/2599) of patients. Like the integrated safety analysis, the most common drug-related adverse event with PA was vomiting (4.2% [95% CI 3.5, 5.0; 109/2599]) (Table 6, Additional file 4).

Most adverse events were of mild-to-moderate severity with 10 grade 3 adverse events reported for 0.3% (95% CI 0.2, 0.6; 8/2599) of patients, and no grade 4 adverse events (Additional file 3). There were 13 serious adverse events in 11 patients (Table 7), of which three were considered drug related: two cases of anaemia and one of epistaxis. In CANTAM, patients with baseline ALT/AST elevations were not excluded from treatment and 1.5% (39/2586) of patients had ALT values > 1.5×ULN, and 6.8% (175/2585) had AST values > 1.5×ULN before PA treatment (Additional file 6). However, there were no reports of symptomatic hepatotoxicity following PA treatment and post-baseline laboratory investigations for hepatotoxicity were therefore not clinically indicated.

### Efficacy

Study SP-C-003-05 was a non-comparative dose-escalation study and included 15 patients aged between 2 and 10 years with uncomplicated *P. falciparum* malaria who received PA granules. Day 28 ACPR in the PP population was 100% (14/14) [10].

**Fig. 4:**
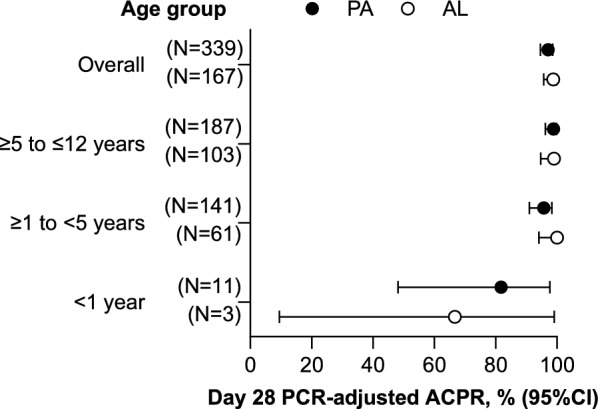
Study SP-C-007-07: day 28 PCR-adjusted ACPR by age (PP population) [11]. *ACPR* adequate clinical and parasitological response, *AL* artemether–lumefantrine, *PA* pyronaridine–artesunate, *PCR* polymerase chain reaction, *PP* per-protocol

In study SP-C-007-07, 535 patients < 12 years with uncomplicated *P. falciparum* malaria were randomized in a 2:1 ratio to receive PA granules (N = 355) or AL crushed tablets (N = 180) [11]. Findings have been published previously but key results are summarized here [11]. Overall, 43.4% (232/535) of patients were aged < 5 years (PA, N = 160; AL, N = 72), with 15 patients aged < 1 year (PA, N = 12; AL, N = 3). All patients had a body weight of < 25 kg. For the primary efficacy endpoint evaluated in the PP population, 97.1% (95% CI 94.6, 98.6; 329/339) of patients receiving PA had PCR-adjusted ACPR on day 28 compared with 98.8% (95% CI 95.7, 99.9; 165/167) of those receiving AL: treatment difference − 1.8 (95% CI − 4.3, 1.6; *P* = 0.22) [11]. Analysis of the primary efficacy outcome by age group showed similar efficacy in children > 1 year old, but lower efficacy for both PA and AL in children < 1 year old, though numbers were small (Fig. 4) [11]. In the ITT population, day 28 PCR-adjusted ACPR was 93.8% (95% CI 90.8, 96.1; 333/355) with PA and 92.8% (95% CI 88.0, 96.1; 167/180) with AL: treatment difference 1.0 (95% CI − 3.2, 6.2; *P* = 0.65) [11].

**Fig. 5:**
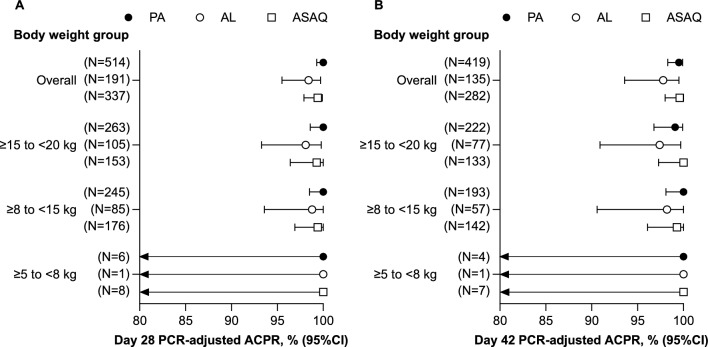
WANECAM (SP-C-013-11): PCR-adjusted ACPR by body weight (PP population). **A** Day 28 PCR-adjusted ACPR. **B** Day 42 PCR-adjusted ACPR. *ACPR* adequate clinical and parasitological response, *AL* artemether–lumefantrine, *ASAQ* artesunate–amodiaquine, *PA* pyronaridine–artesunate, *PCR* polymerase chain reaction, *PP* per-protocol

The primary objective of the WANECAM study was to provide additional efficacy and safety data on PA repeated treatment of recurrent malaria episodes [12]. The full data set including the combined PA tablet and granule efficacy has been previously published [12]. A prospectively planned sub-analysis was conducted including all children with body weight ≥ 5 to < 20 kg presenting with uncomplicated *P. falciparum* malaria who received PA granules, AL dispersible tablets, or ASAQ dissolved tablets. Overall, 556 patients received PA, 233 AL and 366 ASAQ (Additional file 7). In the day 28 PP population, the mean age of children receiving PA was 4.1 (SD 1.8) years, AL 4.4 (1.8) years and ASAQ 3.9 (1.9) years and mean body weight was 14.6 (3.1), 15.0 (2.8) and 14.2 (3.2), respectively (Additional file 8). For the first malaria episode, day 28 PCR-adjusted ACPR was 100% (95% CI 99.3, 100; 514/514) for PA, 98.4% (95% CI 95.5, 99.7; 188/191) for AL, and 99.4% (95% CI 97.9, 99.9; 335/337) for ASAQ (Fig. 5A). Day 42 PCR-adjusted ACPR was 99.5% (95% CI 98.3, 99.9; 417/419) for PA, 97.8% (95% CI 93.6, 99.5; 132/135) for AL, and 99.6% (95% CI 98.0, 100; 281/282) for ASAQ (Fig. 5B). High efficacy was maintained for all treatments at day 28 and day 42 across all body weight categories, though data for patients weighing < 8 kg were limited (Fig. 5).

### Effectiveness

**Fig. 6:**
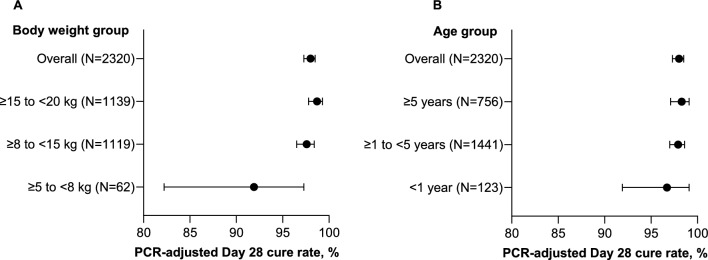
CANTAM (SP-C-021-15): PA granules day 28 PCR-adjusted cure rate (PP population). **A** Day 28 PCR-adjusted cure rate by body weight. **B** Day 28 PCR-adjusted cure rate by age. *PA* pyronaridine–artesunate, *PCR* polymerase chain reaction, *PP* per-protocol

In the real-world study CANTAM, PA clinical effectiveness was evaluated as PCR-adjusted cure at day 28 without an earlier assessment. A post-hoc sub-analysis of clinical cure in all patients with body weight ≥ 5 to < 20 kg presenting with uncomplicated *P. falciparum* malaria was conducted. Overall, the PCR-adjusted cure rate at day 28 in the PP population was 98.0% (95% CI 97.3, 98.5; 2273/2320) and 89.0% (95% CI 87.7, 90.2; 2299/2584) in the ITT population. Cure rates were highest in patients with body weight ≥ 8 kg, with a trend for lower clinical effectiveness in patients with body weight < 8 kg, though the number of patients was low, and 95% CIs were wide (Fig. 6A). High cure rates were observed across all age groups, ranging from 98.3% (95% CI 97.1, 99.1; 743/756) in children ≥ 5 years of age to 96.7% (95% CI 91.9, 99.1; 119/123) in children < 1 year old (Fig. 6B).

## Discussion

An individual patient level integrated safety analysis was conducted to compare the safety and tolerability of PA paediatric granules versus AL for the treatment of uncomplicated malaria. Both treatments were generally well tolerated. The good tolerability of PA granules was confirmed in a real-world study. Data for PA granules from randomized clinical trials showed high anti-malarial efficacy, like that observed for AL and ASAQ [12]. The clinical effectiveness of the PA granule formulation was high in the real-world study.

Vomiting following PA granules occurred predominantly following the first dose of the first treatment, and redosing was usually successful for both drugs. The overall rate of vomiting occurring at any time in the integrated safety analysis was 7.8% (95% CI 6.0, 10.1; 52/667) for PA and 3.4% (95% CI 1.9, 5.8; 12/358; 12/358) for AL whereas in the real-world study CANTAM, it was 5.4% (95% CI 4.6, 6.4; 141/2599) for PA, indicating that this adverse event can be adequately managed in normal clinical practice. For perspective, in a recent meta-analysis of 18 clinical trials conducted in African children aged 6 months to 10 years, vomiting was reported for 10.0% (391/3912) of patients receiving AL and 9.8% (467/4877) of patients receiving DHA–PQP (*P* = 0.24) [20]. The rate of vomiting with PA decreased on the second and third doses, most likely because of improvements in malaria symptoms. The rate of vomiting also declines on repeated treatment [10], which may reflect increased patient acceptance with repeated exposure. Nevertheless, this adverse event appears manageable in the real-world setting, with vomiting associated with the first dose of treatment and with successful re-dosing and no impact on adherence.

When examining the effect of body weight on the rate of vomiting, there were too few patients with body weight < 8 kg in the integrated safety analysis to draw any firm conclusions for PA or AL. However, in CANTAM, vomiting in children weighing < 8 kg occurred at a lower rate with PA (5.7% [95% CI 2.2, 13.8; 4/70]) than in those weighing ≥ 8 to < 15 kg (7.9% [95% CI 6.5, 9.5]; 100/1268), and was lowest in children weighing ≥ 15 to < 20 kg (2.9% [95% CI 2.1, 4.0; 37/1261]), so vomiting did not appear to be directly related to body weight in the real-life setting. This may be because children in the mid-ranges of weight could be less compliant and resist taking medicines, compared with younger and older children; note that vomiting and spitting out the drug were not differentiated in the studies. Vomiting rates were lowest in the heaviest patients in CANTAM, and this was also observed in the integrated safety analysis for both PA (2.9% [95% CI 1.6, 5.3; 10/341]) and AL (2.1% [95% CI 0.8, 5.3; 4/190]). This is consistent with the overall WANECAM study population, including adults and children aged > 6 months receiving paediatric formulations or tablets [12], in which the rate of vomiting in patients weighing < 20 kg was 5.9% (34/572) for PA, 1.5% (5/332) for AL, 6.5% (35/541) for ASAQ and 4.8% (29/608) for DHA–PQP, whereas in patients with body weight ≥ 20 kg, vomiting was less frequent, occurring in 0.5% (4/700) with PA, 0.9% (6/635) with AL, 3.7% (19/520) with ASAQ, and 1.9% (14/732) with DHA–PQP (data on file Medicines for Malaria Venture, SP-C-013-11). Most studies of anti-malarial therapy in children do not report adverse events by body weight, and these findings suggest that this aspect should be given more attention, with data from children < 8 kg particularly required.

Transient asymptomatic increases in liver enzymes have been observed with PA in adults and children and were closely monitored throughout the clinical trial programme [6–13, 15, 17]. Liver enzyme elevations are common in malaria patients and following treatment with anti-malarial therapy [6–13, 15, 17]. A systematic meta-analysis of data from eight randomized clinical trials in adults and children indicated that ALT > 5×ULN was more frequent with PA versus comparators (relative risk 3.34, 95% CI 1.6 to 6.8), though there was no difference for elevated AST > 5×ULN (relative risk 1.80, 95% CI 0.89, 3.7), or bilirubin > 2.5×ULN (relative risk 1.03, 95% CI 0.49 to 2.2) [21]. Note that none of the cases of increased ALT/AST and/or increased total bilirubin so far described following PA treatment had clinical symptoms and all resolved spontaneously. Moreover, the WANECAM study demonstrated that the risk of elevated ALT and/or AST was diminished on retreatment [12]. Here, in the integrated safety analysis of children receiving PA granules, the frequency of increased ALT/AST or total bilirubin was no different from that observed with AL. The rate of potential Hy’s Law cases was also similar, 0.3% (95% CI 0.1, 1.1; 2/650) in the PA group and 0.9% (95% CI 0.2, 2.5; 3/349) in the AL group, relative risk 0.36 (95% CI 0.072, 1.8) (*P* = 0.35). Both potential Hy’s Law cases who received PA had concomitant therapy that is known by itself to be associated with increased hepatic enzymes. The CANTAM study enrolled patients without prior liver function testing, including those that were retrospectively shown to have elevated baseline ALT/AST values [15]. Across the CANTAM study population, including children who received PA granules as reported here, there were no cases where clinical signs and symptoms were observed or prompted biochemical assessments for drug-induced liver injury [15].

The different studies examined in this paper had different primary efficacy endpoints. However, PCR-adjusted ACPR in the PP population was reported for all the randomized clinical trials and values for PA were high and similar to comparators. There was a trend for lower efficacy for PA and AL in patients < 1 year old in study SP-C-007-07, but patient numbers were low, and the 95% CIs were wide. This trend by age was not observed when evaluating clinical effectiveness in study CANTAM. However, in CANTAM, there was a trend for lower effectiveness in children with body weight < 8 kg, but again the number of patients was relatively low, and the 95% CIs were wide, overlapping with values for patients with body weight ≥ 8 kg. This trend by body weight was not observed in WANECAM, but very few patients were enrolled with body weight < 8 kg. Overall, PA efficacy and effectiveness were high, and appear to be maintained across the full range of age and body weight, though further data from children < 1 year old or < 8 kg body weight are needed.

The main limitation of this analysis is that the studies considered had different designs. For the integrated safety analysis, the data for the patient population were harmonized based on body weight, though there were still some variations in drug dosing and observations that could affect the results. Also, most patients were from the WANECAM study. The efficacy analysis considers each study separately, and comparisons cannot be inferred between studies.

## Conclusion

Children are most at risk of malaria but can find tablets difficult to take. Also, ensuring appropriate dosing by body weight can be challenging with tablets. Thus, it is important that paediatric formulations of highly efficacious anti-malarial drugs are available and accessible. For PA, both the adult tablet formulation and the paediatric granule formulation were developed concurrently. Not only was this an efficient strategy in terms of the resources required to complete the development programme, but it promoted access to a paediatric formulation as soon as possible after market authorization was granted. This paper brings together the key safety and efficacy findings for PA paediatric granules. In both randomized clinical trials and in a real-world study, PA paediatric granules were well tolerated, had good efficacy which translated into high clinical effectiveness, and are a valuable additional tool for the treatment of acute uncomplicated malaria in children.

### Supplementary Information

**Additional file 1.** Statistical analysis plan for the integrated safety analysis.

**Additional file 2.** Incidence of all adverse events of any cause by MedDRA primary system organ class and preferred term in the integrated safety analysis of SP-C-003-05, SP-C-007-07, and WANECAM (SP-C-013-11) comparing PA with AL. Results for the PA real-world study CANTAM (SP-C-021-15) are also shown.

**Additional file 3.** Severity of adverse events and incidence of Grade 3 and 4 adverse events in the integrated safety analysis of SP-C-003-05, SP-C-007-07, and WANECAM (SP-C-013-11) comparing PA and AL. Results for the PA real world cohort event monitoring study CANTAM (SP-C-021-15) are also shown.

**Additional file 4.** Incidence of drug-related adverse events by MedDRA primary system organ class and preferred term in the integrated safety analysis of SP-C-003-05, SP-C-007-07, and WANECAM (SP-C-013-11) comparing PA with AL. Results for the PA real-world study CANTAM (SP-C-021-15) are also shown.

**Additional file 5.** Changes from baseline for haematology and biochemistry in the integrated safety analysis of SP-C-003-05, SP-C-007-07, and WANECAM (SP-C-013-11).

**Additional file 6.** Frequency of baseline elevations in alanine aminotransferase and aspartate aminotransferase in CANTAM (SP-C-021-15).

**Additional file 7.** WANECAM (SP-C-013-11) patient disposition.

**Additional file 8.** WANECAM (SP-C-013-11) baseline characteristics (day 28 per protocol population).

## Data Availability

All relevant data are provided in the manuscript or available from published materials as cited. De-identified individual patient data are available from mmv.org on reasonable request for at least 5 years after publication of this article.

## Abbreviations

ACPR: Adequate clinical and parasitological response
AL: Artemether–lumefantrine
ALT: Alanine aminotransferase
ASAQ: Artesunate–amodiaquine
AST: Aspartate aminotransferase
DHA: Dihydroartemisinin
ECG Electrocardiograph ITT: Intention-to-treat
NA: Not applicable
PA: Pyronaridine–artesunate
PCR: Polymerase chain reaction
PP: Per protocol
PQP: Piperaquine (phosphate)
ULN: Upper limit of normal
WHO: World Health Organization
