# Supplementary material for: Safety and efficacy of pyronaridine–artesunate paediatric granules in the treatment of uncomplicated malaria in children: insights from randomized clinical trials and a real-world study
Source: Malar J. 2024 Feb 28;23:61. doi: 10.1186/s12936-024-04885-3 (PMC10902982; doi:10.1186/s12936-024-04885-3)
Supplement: Supplementary file 1 — Additional file 1. Statistical analysis plan for the integrated safety analysis. [file 12936_2024_4885_MOESM1_ESM.pdf]

DATAMAP

STATISTICAL ANALYSIS PLAN FOR PYRAMAX ISS GRANULE SUBMISSION

Author	Martina Wibberg
Version	Final 1.0
Date of version	18-Jun-14

Table of Contents

1. INTRODUCTION 4
2. DEFINITIONS AND DATA CONVENTIONS 4
3. ANALYSIS POPULATION 4
4. PATIENT DISPOSITION 4
5. DEMOGRAPHIC DATA AND BASELINE CHARACTERISTICS 5
7. EXPOSURE TO STUDY DRUG / CONCOMITANT TREATMENTS 5
8. SAFETY ANALYSIS 5
   8.1 Adverse events 5
   8.2 Clinical laboratory data 6
   8.3 Other data related to safety 7
9. LIST OF TABLES AND FIGURES 7
10. LIST OF DATA LISTINGS AND STATISTICAL TABULATIONS 7
11. SIGNATURES 8
12. TABLE SHELLS 9
ULN = Upper limit of normal range 22

Document history

Version	Date	Changes to previous version
1.0	18-Jun-14	

1. INTRODUCTION

This analysis plan describes the ISS analyses planned for the Pyramax paediatric (granule) formulation submission to EMA.

For the analysis of paediatric safety data the safety data of the following studies will be pooled:

- SP-C-003-05, Cohort D, patients treated with the PA granule formulation weighing <20 kg
- SP-C-007-07 (both PA and AL) weighing <20 kg
- WANECAM paediatric patients (weighing <20 kg) from the corresponding sub study analysis, treatment episode 1 only.

All analyses will be performed by DATAMAP GmbH, Freiburg, Germany using SAS®, Version 9.3 in a UNIX environment.

2. DEFINITIONS AND DATA CONVENTIONS

<u>Baseline</u>

Baseline for analysis purpose will be defined as the last measurement prior to first study drug administration, i.e., in general the Day 0 pre-dose assessment.

<u>Study days</u>

Study days will be calculated as actual date minus date of first study drug intake, i.e. the day of first study drug intake will be defined as Day 0.

<u>Calculation of age</u>

Age will be calculated from the date of the screening visit and the date of birth and presented as integer value. If the date of birth is not available age as entered on the CRF will be used.

The following SAS code will be used to calculate age where &dob is the date of birth and &dat is the date of visit 1

floor((intck('MONTH',&dob,&dat)-(day(&dat)<day(&dob)))/12)

<u>Calculation of Body Mass Index (BMI)</u>

BMI will be calculated as weight (kg) / [height (m)]^2

BMI will be rounded to one decimal.

<u>Labelling of treatment groups</u>

Treatment groups will be labelled

Pyronaridine artesunate (if necessary, the abbreviation PA will be used)

Artemether lumefantrine (if necessary, the abbreviation AL will be used)

3. ANALYSIS POPULATION

All patients from the above studies who received at least one dose of study drug will be included in the paediatric safety population.

4. PATIENT DISPOSITION

Patient disposition will be summarised with the number and percentage of patients who were randomized, treated, who discontinued the study prematurely by treatment group and overall. Further, the reasons for premature discontinuation from the study will be summarised.

5. DEMOGRAPHIC DATA AND BASELINE CHARACTERISTICS

Demographic data and baseline characteristics will be presented by treatment group.

Data will be summarised with number of observations, mean, standard deviation, minimum, median, quartiles, and maximum for continuous variables and with number and percentage of patients for categorical variables.

Continuous variables: age, height, weight, BMI

Categorical variables: sex, ethnicity, age category (<=6 months, >6 months to <1 year, 1 to 2 years, 3 to 5 years, >=6 years), body weight category (5-<8 kg, 8-<15 kg, 15-<20 kg).

7. EXPOSURE TO STUDY DRUG / CONCOMITANT TREATMENTS

The following will be summarized:

- Number and percentage of patients who received one, two, three, or four doses of PA, or one, two, three, four, five, six, seven doses of AL. For vomited doses that were repeated the sum of both doses will be used.
- For patients in the PA group: Number and percentage of patients who vomited the first dose, the second dose, or the third dose.
- For patients in the AL group: Number and percentage of patients who vomited the first dose, the second dose, the third dose, the fourth dose, the fifth dose, or the sixth dose.
- Number and percentage of patients who received a repeated dose for the first dose and who vomited the repeated first dose.
- The total amount of dose of study drug administered expressed in mg/kg body weight, separately for artesunate, pyronaridine tetraphosphate, artemether, and lumefantrine. To calculate this, the total number of sachets/tablets taken by a patient will be multiplied by the strength and divided by the patient’s body weight at baseline. For vomited doses that were repeated both the original and the repeated dose will be used.

8. SAFETY ANALYSIS

8.1 Adverse events

The following adverse event summaries will be generated by treatment group.

- An overview of the number and percentage of patients with
  - any adverse event
  - any serious adverse event
  - any severe or life-threatening adverse event
  - any adverse event considered to be related to study drug, whereby related will be defined as possible, probable, definite, or missing relationship, as assessed by the investigator. If the relationship to study medication is missing the worst case will be assumed, i.e. such AEs will also be considered study drug related.
  - any serious adverse event considered to be related to study drug
  - any adverse event leading to death.
- Number and percentage of patients with adverse events by MedDRA primary system organ class and preferred term by treatment group
- Number and percentage of patients with study drug related adverse events (defined as possible, probable, definite or missing relationship to study drug) by MedDRA primary system organ class and preferred term by treatment group
- Number and percentage of patients with serious adverse events by MedDRA primary system organ class and preferred term by treatment group

- Number and percentage of patients with adverse events by MedDRA primary system organ class, preferred term and maximal severity, by treatment group

Listings of all serious adverse events will be provided.

The overview of AEs, AEs by primary system organ class and preferred term, AEs considered to be drug related by primary system organ class and preferred term, serious AEs and serious AEs considered to be drug-related will further be summarized by body weight category and treatment.

Potential differences between PA and AL in AE incidence rates will be analysed using Fisher's exact test.

8.2 Clinical laboratory data

Clinical laboratory data (AST, ALT, total bilirubin, direct bilirubin, if available, alkaline phosphatase, serum creatinine, haemoglobin, platelet count, white blood count, neutrophils, lymphocytes, eosinophils) will be summarised by treatment group and time point, including changes from Day 0 (pre-dose) with the number of observations, mean, standard deviation, median, quartiles, minimum and maximum.

SI values will be used for summarisation.

To evaluate potential anemia, incidence rates of patients having a hemoglobin change from baseline:

≥0 g/L, -20 - < 0 g/L, or < -20 g/L will be summarized for each time point.

Incidence rates of liver enzyme abnormalities will be summarized by time point and for the worst (highest) value based on the following event criteria (note that criteria are not mutually exclusive):

Parameter	Criterion
ALT	≤1.5 ULN, >1.5 ULN and ≤3 x ULN<br>>3xULN; >5xULN; >10xULN
AST	≤1.5 ULN, >1.5 ULN and ≤3 x ULN<br>>3xULN; >5xULN; >10xULN
ALT or AST	>3xULN; >5xULN; >10xULN
Total bilirubin (TBL)	>1.5xULN, >2xULN, >3xULN
ALT or AST & TBL	ALT or AST>3xULN & TBL >2xULN (Hy's Law)

ULN=upper limit of normal range of the local laboratory.

For a combined criterion to be fulfilled all criteria have to be fulfilled at the same time point.

Shift tables of the above categories at Day 0 versus the worst post baseline value will be generated.

The following graphical displays of liver function test data will be generated:

Comparison between treatment groups and between first and subsequent dosing using scatter plots of the peak total bilirubin value versus the peak ALT/AST values (expressed relative to the upper limit of normal) from Day 3 until Day 28 and from Day 7 until Day 28 (E-Dish graphs).

Scatter plots for ALT, total bilirubin showing individual data by day (y axis will present value, x-axis will present Day 0, 3, 7 and 28) – separately for treatment groups for all patients and for patients who had at least one re-treatment.

ALT classifications relative to the normal ranges, incidence rates of potential liver abnormalities and shifts of ALT categories will further be summarised by body weight category.

Potential differences between PA and AL of ALT and AST classifications relative to the normal ranges will be analysed using the Chi-square test and incidence rates of potential liver abnormalities using Fisher's exact test.

8.3 Other data related to safety

Vital signs (systolic and diastolic blood pressure, pulse rate, respiratory rate) will be summarised by treatment and time point, including changes from Day 0 (pre-dose) with the number of observations, mean, standard deviation, median, quartiles, minimum and maximum. Vital signs measured in any position (sitting, supine, standing) will be combined for the summary tables.

9. LIST OF TABLES AND FIGURES

see Section 12

10. LIST OF DATA LISTINGS AND STATISTICAL TABULATIONS

see Section 12

11. SIGNATURES

Author: [redacted]

Approval:

12. TABLE SHELLS

Table 2.7.4-1 Patient disposition and exposure
Paediatric safety population
(Page 1 of 1)

	Pyronaridine artesunate	Artemether lumefantrine	Total
Patients treated (at least one dose)	xxx (100.0)	xxx (100.0)	xxx (100.0)
Patients who completed study	xxx (xx.x)	xxx (xx.x)	xxx (xx.x)
Reason for withdrawal			
Treatment failure	xxx (xx.x)	xxx (xx.x)	xxx (xx.x)
Adverse event	xxx (xx.x)	xxx (xx.x)	xxx (xx.x)
Death	xxx (xx.x)	xxx (xx.x)	xxx (xx.x)
Protocol violation	xxx (xx.x)	xxx (xx.x)	xxx (xx.x)
Lost to follow-up	xxx (xx.x)	xxx (xx.x)	xxx (xx.x)
Withdrawal of consent	xxx (xx.x)	xxx (xx.x)	xxx (xx.x)
Pregnancy	xxx (xx.x)	xxx (xx.x)	xxx (xx.x)
Study terminated by Sponsor	xxx (xx.x)	xxx (xx.x)	xxx (xx.x)
Other	xxx (xx.x)	xxx (xx.x)	xxx (xx.x)

Note: Percentages are based on the number of randomised patients.
For SP-C-013-11 a patient was considered to have completed if the first treatment episode was completed.

Table 2.7.4-2 Demographic characteristics
Paediatric safety population
(Page 1 of 3)

Variable/<br>Statistic/Category	Pyronaridine artesunate (N=xxx)	Artemether lumefantrine (N=xxx)
Gender, n (%)		
Male	xxx (xx.x)	xxx (xx.x)
Female	xxx (xx.x)	xxx (xx.x)
Age (years)		
Available observations	xxx	xxx
Mean	xx.x	xx.x
Standard deviation	x.xx	x.xx
Minimum	xx	xx
Q1	xx.x	xx.x
Median	xx	xx
Q3	xx.x	xx.x
Maximum	xx	xx
Age category, n (%)		
<=6 months	xxx (xx.x)	xxx (xx.x)
>6 months – <1 year	xxx (xx.x)	xxx (xx.x)
1-2 years	xxx (xx.x)	xxx (xx.x)
3-5 years	xxx (xx.x)	xxx (xx.x)
>=6 years	xxx (xx.x)	xxx (xx.x)

Missing values were not included in the calculation of percentages.

Table 2.7.4-2 Demographic characteristics
Paediatric safety population
(Page 2 of 3)

Variable/<br>Statistic/Category	Pyronaridine artesunate (N=xxx)	Artemether lumefantrine (N=xxx)
Height (cm)		
Available observations	xxx	xxx
Mean	xxx.x	xxx.x
Standard deviation	xx.xx	xx.xx
Minimum	xxx	xxx
Q1	xxx	xxx
Median	xxx.x	xxx.x
Q3	xxx	xxx
Maximum	xxx	xxx
Body weight (kg)		
Available observations	xxx	xxx
Mean	xx.x	xx.x
Standard deviation	x.xx	x.xx
Minimum	xx.x	xx.x
Q1	xx.x	xx.x
Median	xx.x	xx.x
Q3	xx.x	xx.x
Maximum	xx.x	xx.x

Missing values were not included in the calculation of percentages.

Table 2.7.4-2 Demographic characteristics
Paediatric safety population
(Page 3 of 3)

Variable/<br>Statistic/Category	Pyronaridine artesunate (N=xxx)	Artemether lumefantrine (N=xxx)
Body weight category, n (%)		
<8 kg	xxx (xx.x)	xxx (xx.x)
8 - <15 kg	xxx (xx.x)	xxx (xx.x)
15 - <20 kg	xxx (xx.x)	xxx (xx.x)
Body mass index (kg/m**2)		
Available observations	xxx	xxx
Mean	xx.xx	xx.xx
Standard deviation	x.xxx	x.xxx
Minimum	xx.x	xx.x
Q1	xx.xx	xx.xx
Median	xx.xx	xx.xx
Q3	xx.xx	xx.xx
Maximum	xx.x	xx.x

Missing values were not included in the calculation of percentages.

Table 2.7.4-3 Study drug exposure
Paediatric safety population
(Page 1 of 1)

Variable/ Category	Pyronaridine artesunate (N=xxx) n (%)	Artemether lumefantrine (N=xxx) n (%)
Total number of doses taken *		
One	xxx (xx.x)	xxx (xx.x)
Two	xxx (xx.x)	xxx (xx.x)
Three	xxx (xx.x)	xxx (xx.x)
Four	xxx (xx.x)	xxx (xx.x)
Five	xxx (xx.x)	xxx (xx.x)
Six	NA	xxx (xx.x)
Seven	NA	xxx (xx.x)
Eight	NA	xxx (xx.x)
Dose repeated		
Day 0 – Dose 1	xx (xx.x)	xx (xx.x)
Day 0 – Dose 2	NA	xx (xx.x)
Day 1 – Dose 1	xx (xx.x)	xx (xx.x)
. . .		
Dose vomited within 30 minutes		
Day 0 - Dose 1	xxx (xx.x)	xxx (xx.x)
Repeated dose	xxx (xx.x)	xxx (xx.x)
Day 0 - Dose 2	NA	xxx (xx.x)
Repeated dose	NA	xxx (xx.x)
. . .		

NA = not applicable
* Original and repeat doses were taken into account.

Table 2.7.4-4 Study drug dosage
Paediatric safety population
(Page 1 of 1)

Variable/ Statistic	Pyronaridine artesunate (N=xxx)	Artemether lumefantrine (N=xxx)
mg/kg	Artesunate	Artemether
Available observations	xxx	xxx
Mean	x.xx	x.xx
SD	x.xxx	x.xxx
Minimum	x.xx	x.xx
Q1	x.xx	x.xx
Median	x.xx	x.xx
Q3	x.xx	x.xx
Maximum	x.xx	x.xx
mg/kg	Pyronaridine	Lumefantrine
Available observations	xxx	xxx
Mean	x.xx	x.xx
SD	x.xxx	x.xxx
Minimum	x.xx	x.xx
Q1	x.xx	x.xx
Median	x.xx	x.xx
Q3	x.xx	x.xx
Maximum	x.xx	x.xx

Note: Original and repeat doses were taken into account.

Table 2.7.4-5 Overview of adverse events, by treatment
Paediatric safety population
(Page 1 of 1)

Number (%) of patients with	Pyronaridine artesunate n (%)	Artemether lumefantrine n (%)	p-value
Number of patients dosed	xxx (100.0)	xxx (100.0)	
Any adverse event	xxx (xx.x)	xxx (xx.x)	0.xxxx
Any drug-related adverse event *	xxx (xx.x)	xxx (xx.x)	0.xxxx
Any serious adverse event	xxx (xx.x)	xxx (xx.x)	0.xxxx
Any serious drug-related adverse event *	xxx (xx.x)	xxx (xx.x)	0.xxxx
Any severe or life-threatening adverse event	xxx (xx.x)	xxx (xx.x)	0.xxxx
Any adverse event leading to death	xxx (xx.x)	xxx (xx.x)	0.xxxx

Note: * Drug-related = possible, probable, definite or missing relationship to study drug.
p-values from Fisher's exact test for the difference between PA and AL.

Table 2.7.4-6 Incidence of all adverse events by MedDRA primary system organ class and preferred term
Paediatric safety population
(Page 1 of n)

Primary system organ class<br>Preferred term	Pyronaridine artesunate<br>n (%)	Artemether lumefantrine<br>n (%)	p-value
Patients dosed	xxx (100.0)	xxx (100.0)	
At least one adverse event	xxx (xx.x)	xxx (xx.x)	0.xxxx
Primary system organ class 1	xxx (xx.x)	xxx (xx.x)	0.xxxx
Preferred term 1	xxx (xx.x)	xxx (xx.x)	0.xxxx
Preferred term 2	xxx (xx.x)	xxx (xx.x)	0.xxxx
Primary system organ class 2	xxx (xx.x)	xxx (xx.x)	0.xxxx
Preferred term 1	xxx (xx.x)	xxx (xx.x)	0.xxxx
Preferred term 2	xxx (xx.x)	xxx (xx.x)	0.xxxx
etc.			

A patient with more than one adverse event within a primary system organ class is counted only once for that class.
p-values from Fisher's exact test for the difference between PA and AL.

Table 2.7.4-7 Incidence of adverse events considered to be study drug related, by MedDRA primary system organ class and preferred term
Paediatric safety population

Programming note: Same layout as Table 2.7.4-6. Present only AEs that were considered to be study drug-related.

Table 2.7.4-8 Incidence of serious adverse events, by MedDRA primary system organ class and preferred term
Paediatric safety population

Programming note: Same layout as Table 2.7.4-6. Present serious AEs.

Table 2.7.4-9 Incidence of serious adverse events considered to be study drug related, by MedDRA primary system organ class and preferred term
Paediatric safety population

Programming note: Same layout as Table 2.7.4-6. Present serious AEs that were considered to be study drug related.

Table 2.7.4-10 Incidence of all adverse events by MedDRA primary system organ class, preferred term, maximal severity
Paediatric safety population
(Page 1 of n)

Primary system organ class	Preferred term	Maximal severity	Pyronaridine artesunate N (%)	Artemether lumefantrine n (%)
Patients dosed			xxx (100.0)	xxx (100.0)
At least one AE	Total	Total	xxx (xx.x)	xxx (xx.x)
		Mild	xxx (xx.x)	xxx (xx.x)
		Moderate	xxx (xx.x)	xxx (xx.x)
		Severe	xxx (xx.x)	xxx (xx.x)
Primary SOC 1	Total	Total	xxx (xx.x)	xxx (xx.x)
		Mild	xxx (xx.x)	xxx (xx.x)
	Preferred term 1	Total	xxx (xx.x)	xxx (xx.x)
		Moderate	xxx (xx.x)	xxx (xx.x)
etc.				

If a patient reported more than one adverse event within the same category, the worst severity was summarised.
A patient with more than one adverse event within a primary system organ class is counted only once for that class.

Table 2.7.4-11 Summary of ALT values (U/L) by treatment and time point
Paediatric safety population
(Page 1 of n)

	Time point	N	Mean	SD	Minimum	Q1	Median	Q3	Maximum
Pyronaridine artesunate (N=xxx)									
Raw values	Day 0	xxx	xx.x	x.xx	x.xx	x.xx	x.xx	x.xx	x.xx
	Day 3	xxx	xx.x	x.xx	x.xx	x.xx	x.xx	x.xx	x.xx
	Day 7	xxx	xx.x	x.xx	x.xx	x.xx	x.xx	x.xx	x.xx
	Day 28	xxx	xx.x	x.xx	x.xx	x.xx	x.xx	x.xx	x.xx
Changes from Day 0	Day 3	xxx	xx.x	x.xx	x.xx	x.xx	x.xx	x.xx	x.xx
	Day 7	xxx	xx.x	x.xx	x.xx	x.xx	x.xx	x.xx	x.xx
	Day 28	xxx	xx.x	x.xx	x.xx	x.xx	x.xx	x.xx	x.xx
Artemether lumefantrine (N=xxx)									
Raw values	Day 0	xxx	xx.x	x.xx	x.xx	x.xx	x.xx	x.xx	x.xx
	Day 3	xxx	xx.x	x.xx	x.xx	x.xx	x.xx	x.xx	x.xx
	Day 7	xxx	xx.x	x.xx	x.xx	x.xx	x.xx	x.xx	x.xx
	Day 28	xxx	xx.x	x.xx	x.xx	x.xx	x.xx	x.xx	x.xx
Changes from Day 0	Day 3	xxx	xx.x	x.xx	x.xx	x.xx	x.xx	x.xx	x.xx
	Day 7	xxx	xx.x	x.xx	x.xx	x.xx	x.xx	x.xx	x.xx
	Day 28	xxx	xx.x	x.xx	x.xx	x.xx	x.xx	x.xx	x.xx

Table 2.7.4-12 Summary of AST values (U/L) by treatment and time point
Paediatric safety population

Programming note: Same layout as Table 2.7.4-11 for AST

Table 2.7.4-13 Summary of total bilirubin values (umol/L) by treatment and time point
Paediatric safety population

Programming note: Same layout as Table 2.7.4-11 for total bilirubin

Table 2.7.4-14 Summary of alkaline phosphatase values (U/L) by treatment and time point
Paediatric safety population

Programming note: Same layout as Table 2.7.4-11 for alkaline phosphatase

Table 2.7.4-15 Summary of serum creatinine values (umol/L) by treatment and time point
Paediatric safety population

Programming note: Same layout as Table 2.7.4-11 for serum creatinine

Table 2.7.4-16 Incidence of ALT values relative to the normal range by treatment and time point
Paediatric safety population
(Page 1 of n)

Time point		Pyronaridine artesunate (N=xxx) n / total (%)	Artemether lumefantrine (N=xxx) n / total (%)	p-value
Day 0	ALT <=1.5 x ULN	xx / xxx (xx.x)	xx / xxx (xx.x)	0.xxxx
	ALT >1.5 x ULN and <=3 x ULN	xx / xxx (xx.x)	xx / xxx (xx.x)	
	ALT >3 x ULN and <=5 x ULN	xx / xxx (xx.x)	xx / xxx (xx.x)	
	ALT >5 x ULN and <=10 x ULN	xx / xxx (xx.x)	xx / xxx (xx.x)	
	ALT >10 x ULN	xx / xxx (xx.x)	xx / xxx (xx.x)	
Day 3	ALT <=1.5 x ULN	xx / xxx (xx.x)	xx / xxx (xx.x)	0.xxxx
	ALT >1.5 x ULN and <=3 x ULN	xx / xxx (xx.x)	xx / xxx (xx.x)	
	ALT >3 x ULN and <=5 x ULN	xx / xxx (xx.x)	xx / xxx (xx.x)	
	ALT >5 x ULN and <=10 x ULN	xx / xxx (xx.x)	xx / xxx (xx.x)	
	ALT >10 x ULN	xx / xxx (xx.x)	xx / xxx (xx.x)	
Day 7	...			
Day 28	...			
Highest value post Day 0 ...				

ULN = Upper limit of normal range
p-values from Chi-square test for the difference between PA and AL.

Table 2.7.4-17 Incidence of AST values relative to the normal range, by treatment and time point
Paediatric safety population

Programming note: Same layout as Table 2.7.4-16 for AST

Table 2.7.4-18 Incidence of liver enzyme classifications, by treatment and time point
Paediatric safety population
(Page 1 of n)

Time point		Pyronaridine artesunate (N=xxx) n / total (%)	Artemether lumefantrine (N=xxx) n / total (%)	p-value
Day 0	ALT >3 x ULN	xx / xxx (xx.x)	xx / xxx (xx.x)	0.xxxx
	ALT >5 x ULN	xx / xxx (xx.x)	xx / xxx (xx.x)	0.xxxx
	ALT >10 x ULN	xx / xxx (xx.x)	xx / xxx (xx.x)	0.xxxx
	AST >3 x ULN	xx / xxx (xx.x)	xx / xxx (xx.x)	0.xxxx
	AST >5 x ULN	xx / xxx (xx.x)	xx / xxx (xx.x)	0.xxxx
	AST >10 x ULN	xx / xxx (xx.x)	xx / xxx (xx.x)	0.xxxx
	ALT or AST >3 x ULN	xx / xxx (xx.x)	xx / xxx (xx.x)	0.xxxx
	ALT or AST >5 x ULN	xx / xxx (xx.x)	xx / xxx (xx.x)	0.xxxx
	ALT or AST >10 x ULN	xx / xxx (xx.x)	xx / xxx (xx.x)	0.xxxx
	TBIL >1.5 x ULN			
	TBIL >2 x ULN			
	TBIL >3 x ULN			
	Hy's law (ALT or AST >3 x ULN and TBIL >2 x ULN)			
Day 3				
Day 7	...			
Day 28	...			
Highest value post Day 0 ...				

ULN = Upper limit of normal range
TBIL = total bilirubin
p-value from Fisher's exact test for the difference between PA and AL.

Table 2.7.4-19 Shift table of ALT categories from Day 0 pre dose to the worst post dose value, by treatment
Paediatric safety population
(Page 1 of n)

		Number (%) of patients: Day 0					
Treatment		Total	<1.5 x ULN	>1.5-<=3 x ULN	>3-<=5 x ULN	>5-<=10 x ULN	>10 x ULN
Pyronaridine artesunate	Total	xxx (100)	xxx (xx.x)	xxx (xx.x)	xxx (xx.x)	xxx (xx.x)	xxx (xx.x)
	<1.5 x ULN	xxx (xx.x)	xxx (xx.x)	xxx (xx.x)	xxx (xx.x)	xxx (xx.x)	xxx (xx.x)
	>1.5-<=3 x ULN	xxx (xx.x)	xxx (xx.x)	xxx (xx.x)	xxx (xx.x)	xxx (xx.x)	xxx (xx.x)
	>3-<=5 x ULN	xxx (xx.x)	xxx (xx.x)	xxx (xx.x)	xxx (xx.x)	xxx (xx.x)	xxx (xx.x)
	>5-<=10 x ULN	xxx (xx.x)	xxx (xx.x)	xxx (xx.x)	xxx (xx.x)	xxx (xx.x)	xxx (xx.x)
	>10 x ULN	xxx (xx.x)	xxx (xx.x)	xxx (xx.x)	xxx (xx.x)	xxx (xx.x)	xxx (xx.x)
Artemether lumefantrine	Total	xxx (100)	xxx (xx.x)	xxx (xx.x)	xxx (xx.x)	xxx (xx.x)	xxx (xx.x)
	<1.5 x ULN	xxx (xx.x)	xxx (xx.x)	xxx (xx.x)	xxx (xx.x)	xxx (xx.x)	xxx (xx.x)
	>1.5-<=3 x ULN	xxx (xx.x)	xxx (xx.x)	xxx (xx.x)	xxx (xx.x)	xxx (xx.x)	xxx (xx.x)
	>3-<=5 x ULN	xxx (xx.x)	xxx (xx.x)	xxx (xx.x)	xxx (xx.x)	xxx (xx.x)	xxx (xx.x)
	>5-<=10 x ULN	xxx (xx.x)	xxx (xx.x)	xxx (xx.x)	xxx (xx.x)	xxx (xx.x)	xxx (xx.x)
	>10 x ULN	xxx (xx.x)	xxx (xx.x)	xxx (xx.x)	xxx (xx.x)	xxx (xx.x)	xxx (xx.x)

ULN = upper limit of normal range
Patients with a pre-dose and at least on post dose ALT value are included.

Table 2.7.4-20 Shift table of AST categories from Day 0 pre dose to the worst post dose value, by treatment
Paediatric safety population

Programming note: Same layout as Table 2.7.4-19 for AST

Table 2.7.4-21 Summary of haemoglobin values (g/L) by treatment and time point
Paediatric safety population

Programming note: Same layout as Table 2.7.4-11 for haemoglobin

Table 2.7.4-22 Summary of platelet values (103/mm**3) by treatment and time point**
Paediatric safety population

Programming note: Same layout as Table 2.7.4-11 for platelets

Table 2.7.4-23 Summary of white blood cell count values (103/mm**3) by treatment and time point**
Paediatric safety population

Programming note: Same layout as Table 2.7.4-11 for WBC

Table 2.7.4-24 Summary of absolute neutrophils (103/mm**3) by treatment and time point**
Paediatric safety population

Programming note: Same layout as Table 2.7.4-11 for absolute neutrophils

Table 2.7.4-25 Summary of absolute lymphocytes (103/mm**3) by treatment and time point**
Paediatric safety population

Programming note: Same layout as Table 2.7.4-11 for absolute lymphocytes

Table 2.7.4-26 Summary of absolute eosinophils (103/mm**3) by treatment and time point**
Paediatric safety population

Programming note: Same layout as Table 2.7.4-11 for absolute eosinphils

Table 2.7.4-27 Incidence of changes from baseline in haemoglobin
Paediatric safety population
(Page 1 of 1)

Time point	Change from baseline	Pyronaridine artesunate (N=xxx) n (%)	Artemether lumefantrine (N=xxx) n (%)
Day 3	Available observations	xxx (100)	xxx (100)
	< -20 g/L (decrease >20 g/L)	xx (xx.x)	xx (xx.x)
	-20 - <0 g/L (decrease >0-20 g/L)	xx (xx.x)	xx (xx.x)
	>=0 g/L (increase or no change)	xx (xx.x)	xx (xx.x)
Day 7	Available observations	xxx (100)	xxx (100)
	< -20 g/L (decrease >20 g/L)	xx (xx.x)	xx (xx.x)
	-20 - <0 g/L (decrease >0-20 g/L)	xx (xx.x)	xx (xx.x)
	>=0 g/L (increase or no change)	xx (xx.x)	xx (xx.x)
Day 28	Available observations	xxx (100)	xxx (100)
	< -20 g/L (decrease >20 g/L)	xx (xx.x)	xx (xx.x)
	-20 - <0 g/L (decrease >0-20 g/L)	xx (xx.x)	xx (xx.x)
	>=0 g/L (increase or no change)	xx (xx.x)	xx (xx.x)

Percentages are based on the number of available observations at each time point.

Table 2.7.4-28 Summary of vital signs
Paediatric safety population
(Page 1 of n)

Parameter: Systolic blood pressure (mm Hg)

	Time point	N	Mean	SD	Minimum	Q1	Median	Q3	Maximum
Pyronaridine artesunate (N=xxx)									
Raw values	Day 0	xxx	xxx.x	x.xx	xxx	xxx.x	xxx.x	xxx.x	xxx
	Day 1	xxx	xxx.x	x.xx	xxx	xxx.x	xxx.x	xxx.x	xxx
	Day 2	xxx	xxx.x	x.xx	xxx	xxx.x	xxx.x	xxx.x	xxx
	Day 3	xxx	xxx.x	x.xx	xxx	xxx.x	xxx.x	xxx.x	xxx
	Day 7	xxx	xxx.x	x.xx	xxx	xxx.x	xxx.x	xxx.x	xxx
	Day 14	xxx	xxx.x	x.xx	xxx	xxx.x	xxx.x	xxx.x	xxx
	Day 28	xxx	xxx.x	x.xx	xxx	xxx.x	xxx.x	xxx.x	xxx
	Day 35	xxx	xxx.x	x.xx	xxx	xxx.x	xxx.x	xxx.x	xxx
	Day 42	xxx	xxx.x	x.xx	xxx	xxx.x	xxx.x	xxx.x	xxx
Changes from Day 0	Day 1	xxx	xx.x	x.xx	xxx	xxx.x	xxx.x	xxx.x	xxx
	Day 2	xxx	xx.x	x.xx	xxx	xxx.x	xxx.x	xxx.x	xxx
	Day 3	xxx	xx.x	x.xx	xxx	xxx.x	xxx.x	xxx.x	xxx
	Day 7	xxx	xx.x	x.xx	xxx	xxx.x	xxx.x	xxx.x	xxx
	Day 14	xxx	xx.x	x.xx	xxx	xxx.x	xxx.x	xxx.x	xxx
	Day 28	xxx	xx.x	x.xx	xxx	xxx.x	xxx.x	xxx.x	xxx
	Day 35	xxx	xx.x	x.xx	xxx	xxx.x	xxx.x	xxx.x	xxx
	Day 42	xxx	xx.x	x.xx	xxx	xxx.x	xxx.x	xxx.x	xxx

Programming note: Continue with treatment group artemether lumefantrine. Vital signs diastolic blood pressure (mm Hg) and pulse (bpm) will follow on subsequent pages.

Table 2.7.4-29 Overview of adverse events, by body weight category
Paediatric safety population
(Page 1 of 1)

Programming note: Same layout as Table 2.7.4-5. Present body weight categories <8 kg, 8-<15 kg and 15-<20 kg.

Table 2.7.4-30 Incidence of all adverse events by MedDRA primary system organ class and preferred term
Paediatric safety population
(Page 1 of n)

Programming note: Same layout as Table 2.7.4-6. Present body weight categories <8 kg, 8-<15 kg and 15-<20 kg.

Table 2.7.4-31 Incidence of adverse events considered to be study drug related, by MedDRA primary system organ class and preferred term, by body weight category
Paediatric safety population

Programming note: Same layout as Table 2.7.4-30. Present only AEs that were considered to be study drug-related.

Table 2.7.4-32 Incidence of serious adverse events, by MedDRA primary system organ class and preferred term, by body weight category
Paediatric safety population

Programming note: Same layout as Table 2.7.4-30. Present serious AEs.

Table 2.7.4-33 Incidence of serious adverse events considered to be study drug related, by MedDRA primary system organ class and preferred term, by body weight category
Paediatric safety population

Programming note: Same layout as Table2.7.4-30. Present serious AEs that were considered to be study drug related.

Table 2.7.4-34 Incidence of ALT values relative to the normal range by treatment and time point, by body weight category
Paediatric safety population

Programming note: Same layout as Table 2.7.4-16. Present body weight categories <8 kg, 8-<15 kg and 15-<20 kg.

Table 2.7.4-35 Incidence of liver enzyme classifications, by body weight category and time point
Paediatric safety population

Programming note: Same layout as Table 2.7.4-18. Present body weight categories <8 kg, 8-<15 kg and 15-<20 kg.

Table 2.7.4-36 Shift table of ALT categories from Day 0 pre dose to the worst post dose value, by treatment and body weight category
Paediatric safety population

Programming note: Same layout as Table 2.7.4-19. Present body weight categories <8 kg, 8-<15 kg and 15-<20 kg.

Figure 2.7.4-1 Scatter plot of peak bilirubin versus peak ALT >= Day 3
Paediatric safety population
(Page 1 of 1)

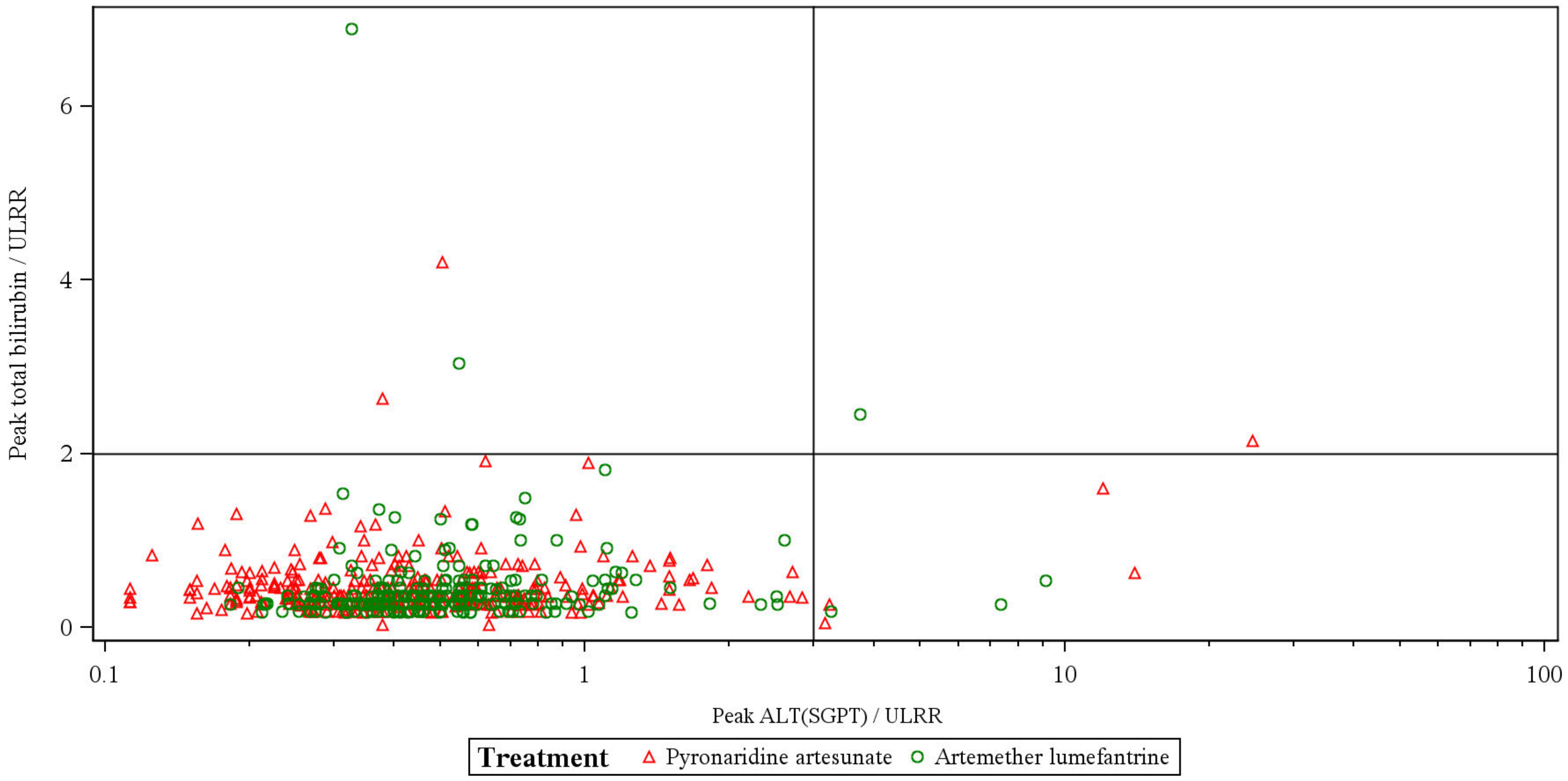

Figure 2.7.4-2 Scatter plot of peak bilirubin versus peak AST >= Day 3
Paediatric safety population

Programming note: same layout as Figure 2.7.4-1

Figure 2.7.4-3 Scatter plot of peak bilirubin versus peak ALT >= Day 7
Paediatric safety population

Programming note: same layout as Figure 2.7.4-1

Figure 2.7.4-4 Scatter plot of peak bilirubin versus peak AST >= Day 7
Paediatric safety population

Programming note: same layout as Figure 2.7.4-1

Figure 2.7.4-5 Scatter plot of ALT over time
Paediatric safety population

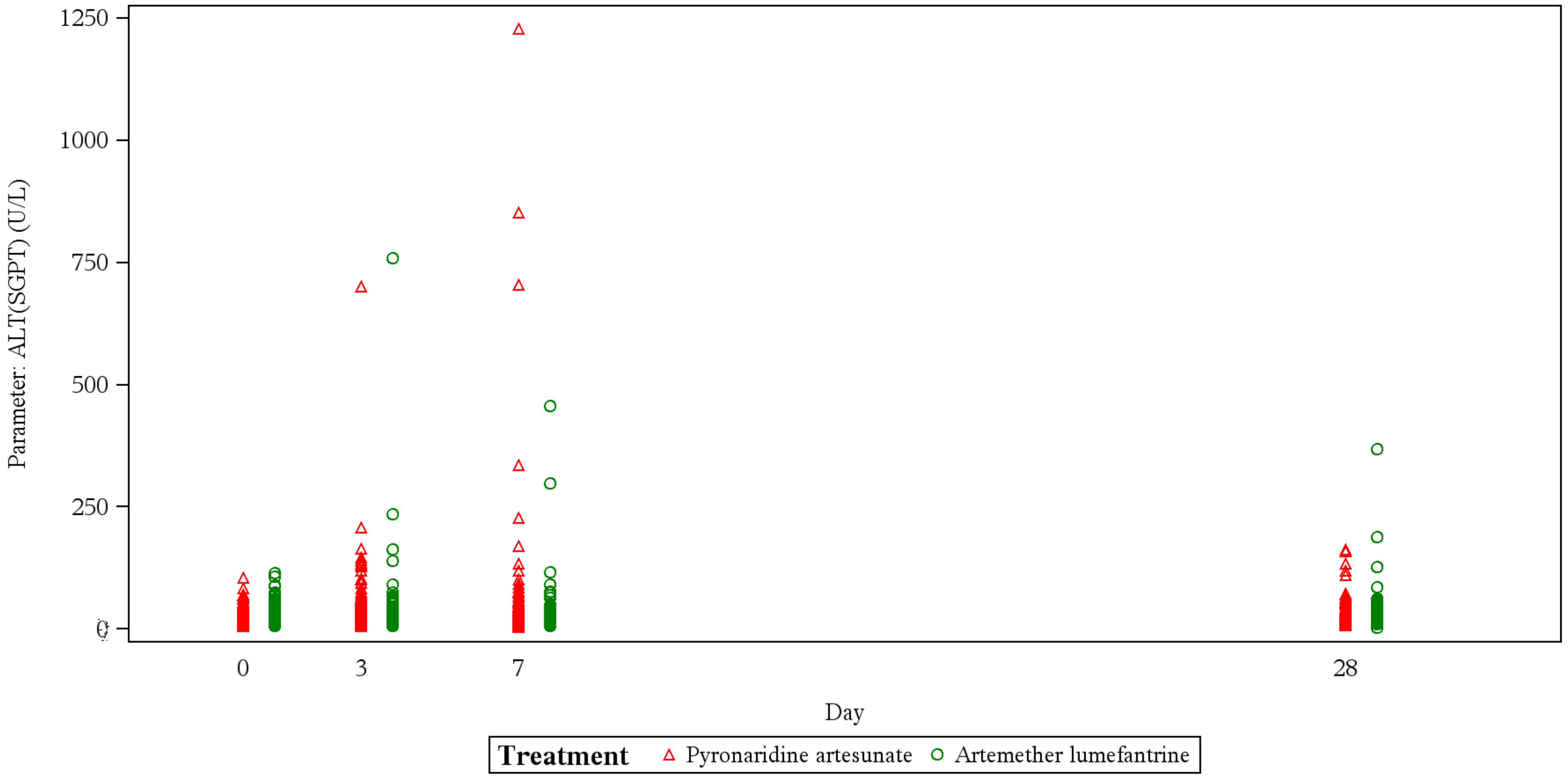

Figure 2.7.4-6 Scatter plot of AST over time
Paediatric safety population

Programming note: this figure will be similar to 2.7.4-5.

Figure 2.7.4-7 Scatter plot of total bilirubin over time
Paediatric safety population

Programming note: this figure will be similar to 2.7.4-5.

Listing 2.7.4-1 Listing of serious adverse events
Paediatric safety population
(Page 1 of 1)

Treatment group: Pyronaridine artesunate

Pat. No.	Age/ Sex/ Weight	Adverse event INVESTIGATOR TERM/ MedDRA preferred term/SOC	Start date/ day	Stop day/ day	Severity	Relation-ship with study drug	Action taken	Outcome
xx-xxxx	8/F/xx.x	XXXXXXXXXXXX/ xxxxxxxxxxxxx/xxxxxxxxx	ddMMMyyyy/xx	ddMMMyyyy/xx	mild	possible	none	resolved
etc.								
