# Supplementary material for: Safety and efficacy of pyronaridine–artesunate paediatric granules in the treatment of uncomplicated malaria in children: insights from randomized clinical trials and a real-world study
Source: Malar J. 2024 Feb 28;23:61. doi: 10.1186/s12936-024-04885-3 (PMC10902982; doi:10.1186/s12936-024-04885-3)
Supplement: Supplementary file 2 — Additional file 2. Incidence of all adverse events of any cause by MedDRA primary system organ class and preferred term in the integrated safety analysis of SP-C-003-05, SP-C-007-07, and WANECAM (SP-C-013-11) comparing PA with AL. Results for the PA real-world study CANTAM (SP-C-021-15) are also shown. [file 12936_2024_4885_MOESM2_ESM.pdf]

Additional file 2. Incidence of all adverse events of any cause by MedDRA primary system organ class and preferred term in the integrated safety analysis of SP-C-003-05, SP-C-007-07, and WANECAM (SP-C-013-11) comparing PA with AL. Results for the PA real-world study CANTAM (SP-C-021-15) are also shown.

Primary system class and preferred term	Integrated safety analysis			SP-C-021-15 (N=2599)
	PA (N=667)	AL (N=358)	*P* value[a]	
Blood and lymphatic system disorders	69 (10.3)	48 (13.4)	0.15	23 (0.9)
Anaemia	35 (5.2)	17 (4.7)	0.77	20 (0.8)
Neutropenia	15 (2.2)	11 (3.1)	0.41	0
Monocytosis	10 (1.5)	10 (2.8)	0.16	0
Splenomegaly	6 (0.9)	4 (1.1)	0.75	2 (0.1)
Leukopenia	4 (0.6)	0	0.30	1 (<0.1)
Thrombocytopenia	4 (0.6)	2 (0.6)	1.00	0
Eosinophilia	3 (0.4)	2 (0.6)	1.00	0
Leukocytosis	1 (0.1)	0	1.00	0
Lymphadenopathy	1 (0.1)	1 (0.3)	1.00	0
Lymphopenia	1 (0.1)	0	1.00	0
Basophilia	0	2 (0.6)	0.12	0
Lymphocytosis	0	3 (0.8)	0.04	0
Neutrophilia	0	1 (0.3)	0.35	0
White blood cell disorder	0	0	NA	1 (<0.1)
Cardiac disorders	0	1 (0.3)	0.35	0
Arrhythmia	0	1 (0.3)	0.35	0
Ear and labyrinth disorders	0	2 (0.6)	0.12	3 (0.1)
Ear pain	0	2 (0.6)	0.12	1 (<0.1)
Ear congestion	0	0	NA	1 (<0.1)
Ear swelling	0	0	NA	1 (<0.1)
Eye disorders	13 (1.9)	5 (1.4)	0.62	0
Conjunctivitis	13 (1.9)	5 (1.4)	0.62	0
Gastrointestinal disorders	83 (12.4)	33 (9.2)	0.15	192 (7.4)
Vomiting	52 (7.8)	12 (3.4)	0.0043	141 (5.4)
Abdominal pain	15 (2.2)	7 (2.0)	0.83	12 (0.5)
Diarrhoea	6 (0.9)	4 (1.1)	0.75	37 (1.4)
Aphthous stomatitis	4 (0.6)	1 (0.3)	0.66	0
Enteritis	3 (0.4)	2 (0.6)	1.00	0
Gastritis	3 (0.4)	1 (0.3)	1.00	0
Constipation	2 (0.3)	3 (0.8)	0.35	1 (<0.1)
Abdominal distension	1 (0.1)	0	1.00	0
Abdominal pain upper	1 (0.1)	0	1.00	0
Colitis	1 (0.1)	1 (0.3)	1.00	0
Dental caries	1 (0.1)	1 (0.3)	1.00	0
Enterocolitis	1 (0.1)	4 (1.1)	0.053	0
Gastric disorder	1 (0.1)	0	1.00	0
Perianal erythema	1 (0.1)	0	1.00	0
Rectal prolapse	1 (0.1)	0	1.00	0
Mouth ulceration	0	1 (0.3)	0.35	1 (<0.1)
Faeces discoloured	0	0	NA	1 (<0.1)

Haematochezia	0	0	NA	1 (<0.1)
Nausea	0	0	NA	2 (0.1)
Oral disorder	0	0	NA	1 (<0.1)
Tongue ulceration	0	0	NA	1 (<0.1)
Tooth disorder	0	0	NA	1 (<0.1)
General disorders and administration site conditions	41 (6.1)	11 (3.1)	0.036	174 (6.7)
Pyrexia	20 (3.0)	7 (2.0)	0.41	140 (5.4)
Influenza like illness	17 (2.5)	4 (1.1)	0.17	14 (0.5)
Asthenia	2 (0.3)	0	0.55	13 (0.5)
Fatigue	2 (0.3)	0	0.55	5 (0.2)
Chest pain	1 (0.1)	0	1.00	0
Hyperpyrexia	1 (0.1)	0	1.00	0
Multi-organ failure	1 (0.1)	0	1.00	0
Swelling	1 (0.1)	0	1.00	0
Chills	0	0	NA	1 (<0.1)
Hyperthermia	0	0	NA	1 (<0.1)
Induration	0	0	NA	1 (<0.1)
Hepatobiliary disorders	1 (0.1)	1 (0.3)	1.00	1 (<0.1)
Drug-induced liver injury	1 (0.1)	0	1.00	0
Hyperbilirubinaemia	1 (0.1)	0	1.00	0
Jaundice	0	1 (0.3)	0.3493	0
Hepatomegaly	0	0	NA	1 (<0.1)
Infections and infestations	230 (34.5)	115 (32.1)	0.4882	79 (3.0)
Bronchitis	93 (13.9)	53 (14.8)	0.7085	9 (0.3)
Rhinitis	38 (5.7)	27 (7.5)	0.2822	2 (0.1)
Upper respiratory tract infection	34 (5.1)	12 (3.4)	0.2104	0
Nasopharyngitis	14 (2.1)	3 (0.8)	0.1984	7 (0.3)
Helminthic infection	9 (1.3)	2 (0.6)	0.3463	0
Infection parasitic	7 (1.0)	1 (0.3)	0.2735	0
Ear infection	6 (0.9)	1 (0.3)	0.4320	1 (<0.1)
Oral herpes	6 (0.9)	3 (0.8)	1.00	2 (0.1)
Respiratory tract infection	6 (0.9)	0	0.0971	3 (0.1)
Tinea capitis	6 (0.9)	0	0.0971	0
Urinary tract infection	6 (0.9)	2 (0.6)	0.7205	1 (<0.1)
Influenza	5 (0.7)	2 (0.6)	1.00	16 (0.6)
Otitis media acute	5 (0.7)	3 (0.8)	1.00	0
Gastroenteritis	4 (0.6)	1 (0.3)	0.6630	2 (0.1)
Malaria	4 (0.6)	1 (0.3)	0.6630	7 (0.3)
Parasitic gastroenteritis	4 (0.6)	1 (0.3)	0.6630	1 (<0.1)
Pharyngitis	4 (0.6)	2 (0.6)	1.00	2 (0.1)
Tinea infection	4 (0.6)	0	0.3041	0
Varicella	4 (0.6)	4 (1.1)	0.4606	1 (<0.1)
Acarodermatitis	3 (0.4)	2 (0.6)	1.00	5 (0.2)
Amoebiasis	3 (0.4)	0	0.5557	0
Ascariasis	3 (0.4)	1 (0.3)	1.00	0
Furuncle	3 (0.4)	2 (0.6)	1.00	0
Impetigo	3 (0.4)	0	0.5557	2 (0.1)
Pneumonia	3 (0.4)	5 (1.4)	0.1361	2 (0.1)
Wound infection	3 (0.4)	0	0.5557	0

Anal fungal infection	2 (0.3)	0	0.5450	0
Body tinea	2 (0.3)	0	0.5450	0
Fungal skin infection	2 (0.3)	1 (0.3)	1.00	0
Gastrointestinal infection	2 (0.3)	0	0.5450	0
Giardiasis	2 (0.3)	0	0.5450	0
Hookworm infection	2 (0.3)	0	0.5450	0
Infection	2 (0.3)	0	0.5450	0
Otitis media	2 (0.3)	2 (0.6)	0.6145	0
Plasmodium falciparum infection	2 (0.3)	0	0.5450	0
Pyoderma	2 (0.3)	0	0.5450	0
Rash pustular	2 (0.3)	2 (0.6)	0.6145	0
Abscess limb	1 (0.1)	0	1.00	1 (<0.1)
Bronchopneumonia	1 (0.1)	0	1.00	0
Cutaneous leishmaniasis	1 (0.1)	0	1.00	0
Enterocolitis fungal	1 (0.1)	1 (0.3)	1.00	0
Fungal infection	1 (0.1)	0	1.00	1 (<0.1)
Gastrointestinal fungal infection	1 (0.1)	0	1.00	0
Mumps	1 (0.1)	0	1.00	0
Oral infection	1 (0.1)	0	1.00	0
Skin infection	1 (0.1)	1 (0.3)	1.00	0
Staphylococcal skin infection	1 (0.1)	1 (0.3)	1.00	1 (<0.1)
Taeniasis	1 (0.1)	0	1.00	0
Tonsillitis	1 (0.1)	3 (0.8)	0.1253	4 (0.2)
Trichomoniasis intestinal	1 (0.1)	1 (0.3)	1.00	0
Acute tonsillitis	0	1 (0.3)	0.3493	0
Dysentery	0	1 (0.3)	0.3493	2 (0.1)
Febrile infection	0	1 (0.3)	0.3493	0
Folliculitis	0	2 (0.6)	0.1218	0
Oral candidiasis	0	2 (0.6)	0.1218	1 (<0.1)
Paronychia	0	1 (0.3)	0.3493	0
Salmonellosis	0	1 (0.3)	0.3493	0
Schistosomiasis	0	2 (0.6)	0.1218	0
Typhoid fever	0	1 (0.3)	0.3493	1 (<0.1)
Abscess	0	0	NA	2 (0.1)
Abscess oral	0	0	NA	1 (<0.1)
Bacterial infection	0	0	NA	2 (0.1)
Bacterial parotitis	0	0	NA	1 (<0.1)
Chest wall abscess	0	0	NA	1 (<0.1)
Measles	0	0	NA	1 (<0.1)
Pulpitis dental	0	0	NA	1 (<0.1)
Sepsis	0	0	NA	1 (<0.1)
Injury, poisoning and procedural complications	10 (1.5)	7 (2.0)	0.6128	0
Wound	4 (0.6)	1 (0.3)	0.6630	0
Limb injury	2 (0.3)	1 (0.3)	1.00	0
Excoriation	1 (0.1)	0	1.00	0
Face injury	1 (0.1)	1 (0.3)	1.00	0
Injury	1 (0.1)	1 (0.3)	1.00	0
Mouth injury	1 (0.1)	0	1.00	0
Forearm fracture	0	1 (0.3)	0.3493	0

Thermal burn	0	2 (0.6)	0.1218	0
Investigations	118 (17.7)	76 (21.2)	0.1810	2 (0.1)
Platelet count increased	30 (4.5)	17 (4.7)	0.8761	0
Blood glucose decreased	27 (4.0)	15 (4.2)	1.00	0
Aspartate aminotransferase increased	26 (3.9)	16 (4.5)	0.7413	0
Electrocardiogram QT prolonged	21 (3.1)	29 (8.1)	0.0007	0
Blood albumin decreased	17 (2.5)	14 (3.9)	0.2523	0
Alanine aminotransferase increased	15 (2.2)	6 (1.7)	0.6475	0
Blood potassium increased	15 (2.2)	4 (1.1)	0.2335	0
Haemoglobin decreased	14 (2.1)	6 (1.7)	0.8138	1 (<0.1)
Haematocrit decreased	13 (1.9)	4 (1.1)	0.4438	0
White blood cell count increased	9 (1.3)	2 (0.6)	0.3463	0
Platelet count decreased	6 (0.9)	1 (0.3)	0.4320	0
Transaminases increased	6 (0.9)	0	0.0971	0
Blood creatinine decreased	5 (0.7)	6 (1.7)	0.2064	0
Electrocardiogram abnormal	3 (0.4)	1 (0.3)	1.00	0
Red blood cell count increased	3 (0.4)	0	0.5557	0
Blood alkaline phosphatase increased	2 (0.3)	2 (0.6)	0.6145	0
Blood creatine phosphokinase increased	2 (0.3)	0	0.5450	0
Blood glucose increased	2 (0.3)	2 (0.6)	0.6145	0
Blood creatinine increased	1 (0.1)	0	1.00	0
Blood potassium decreased	1 (0.1)	0	1.00	0
Blood sodium increased	1 (0.1)	2 (0.6)	0.2806	0
Eosinophil count increased	1 (0.1)	0	1.00	0
Red blood cell count decreased	1 (0.1)	1 (0.3)	1.00	0
White blood cells urine positive	1 (0.1)	0	1.00	0
Blood bilirubin increased	0	3 (0.8)	0.0424	0
Blood urea increased	0	1 (0.3)	0.3493	0
Reticulocyte count increased	0	1 (0.3)	0.3493	0
Weight decreased	0	0	NA	1 (<0.1)
Metabolism and nutrition disorders	12 (1.8)	3 (0.8)	0.2827	15 (0.6)
Decreased appetite	8 (1.2)	1 (0.3)	0.1732	13 (0.5)
Hypercreatininaemia	3 (0.4)	0	0.5557	0
Hyperglycaemia	1 (0.1)	0	1.00	0
Dehydration	0	1 (0.3)	0.3493	2 (0.1)
Hyperkalaemia	0	1 (0.3)	0.3493	0
Musculoskeletal and connective tissue disorders	0	0	NA	1 (<0.1)
Arthalgia	0	0	NA	1 (<0.1)
Nervous system disorders	12 (1.8)	2 (0.6)	0.1564	18 (0.7)
Headache	11 (1.6)	2 (0.6)	0.2395	15 (0.6)
Somnolence	1 (0.1)	0	1.00	1 (<0.1)
Dizziness	0	0	NA	3 (0.1)
Seizure	0	0	NA	1 (<0.1)
Renal and urinary disorders	3 (0.4)	0	0.5557	0
Enuresis	1 (0.1)	0	1.00	0
Ketonuria	1 (0.1)	0	1.00	0

Proteinuria	1 (0.1)	0	1.00	0
Respiratory, thoracic and mediastinal disorders	64 (9.6)	29 (8.1)	0.4940	64 (2.5)
Cough	51 (7.6)	24 (6.7)	0.6170	49 (1.9)
Productive cough	5 (0.7)	2 (0.6)	1.00	0
Rhinorrhoea	3 (0.4)	1 (0.3)	1.00	12 (0.5)
Epistaxis	2 (0.3)	1 (0.3)	1.00	2 (0.1)
Laryngeal pain	2 (0.3)	0	0.5450	0
Lung disorder	2 (0.3)	1 (0.3)	1.00	0
Asthma	1 (0.1)	1 (0.3)	1.00	0
Rales	1 (0.1)	0	1.00	0
Bronchopneumopathy	0	1 (0.3)	0.3493	0
Dyspnoea	0	0	NA	1 (<0.1)
Oropharyngeal pain	0	0	NA	2 (0.1)
Pneumonitis	0	0	NA	1 (<0.1)
Productive cough	0	0	NA	1 (<0.1)
Skin and subcutaneous tissue disorders	23 (3.4)	10 (2.8)	0.7111	25 (1.0)
Dermatosis	4 (0.6)	1 (0.3)	0.6630	1 (<0.1)
Prurigo	4 (0.6)	1 (0.3)	0.6630	0
Eczema	3 (0.4)	0	0.5557	0
Rash	3 (0.4)	2 (0.6)	1.00	4 (0.2)
Skin lesion	2 (0.3)	0	0.5450	0
Blister	1 (0.1)	0	1.00	0
Dermatitis	1 (0.1)	0	1.00	0
Miliaria	1 (0.1)	0	1.00	2 (0.1)
Pruritus	1 (0.1)	2 (0.6)	0.2806	6 (0.2)
Pruritus generalised	1 (0.1)	0	1.00	1 (<0.1)
Rash vesicular	1 (0.1)	0	1.00	0
Skin mass	1 (0.1)	0	1.00	0
Skin necrosis	1 (0.1)	0	1.00	0
Rash pruritic	0	1 (0.3)	0.3493	0
Toxic epidermal necrolysis	0	1 (0.3)	0.3493	0
Urticaria	0	2 (0.6)	0.1218	0
Dermatitis	0	0	NA	3 (0.1)
Hyperhidrosis	0	0	NA	5 (0.2)
Rash pruritic	0	0	NA	1 (<0.1)
Skin swelling	0	0	NA	1 (<0.1)
Stevens-Johnson syndrome	0	0	NA	1 (<0.1)
Swelling face	0	0	NA	1 (<0.1)
Urticaria	0	0	NA	1 (<0.1)

[a] Pyronaridine-artesunate (PA) versus artemether-lumefantrine (AL); NA, not applicable.
Values, are n (%).
