# Supplementary material for: Safety and efficacy of pyronaridine–artesunate paediatric granules in the treatment of uncomplicated malaria in children: insights from randomized clinical trials and a real-world study
Source: Malar J. 2024 Feb 28;23:61. doi: 10.1186/s12936-024-04885-3 (PMC10902982; doi:10.1186/s12936-024-04885-3)
Supplement: Supplementary file 3 — Additional file 3. Severity of adverse events and incidence of Grade 3 and 4 adverse events in the integrated safety analysis of SP-C-003-05, SP-C-007-07, and WANECAM (SP-C-013-11) comparing PA and AL. Results for the PA real world cohort event monitoring study CANTAM (SP-C-021-15) are also shown. [file 12936_2024_4885_MOESM3_ESM.pdf]

**Additional file 3. Severity of adverse events and incidence of Grade 3 and 4 adverse events in the integrated safety analysis of SP-C-003-05, SP-C-007-07, and WANECAM (SP-C-013-11) comparing PA and AL. Results for the PA observational study CANTAM (SP-C-021-15) are also shown.**

| Severity grade | Integrated safety analysis | | | | | SP-C-021-15 (N=2599) | |
|---|---|---|---|---|---|---|---|
| | PA (N=667) | | AL (N=358) | | *P* value[a] | | |
| | N | % (95%CI) | N | % (95%CI) | | N | % (95%CI) |
| Total | 426 | 63.9 (60.2, 67.4) | 222 | 62.0 (56.9, 66.9) | 0.59 | 460 | 17.7 (16.3, 19.2) |
| Grade 1 | 299 | 44.8 (41.1, 48.6) | 178 | 49.7 (44.6, 54.9) | 0.15 | 244 | 9.4 (8.3, 10.6) |
| Grade 2 | 117 | 17.5 (14.8, 20.6) | 40 | 11.2 (8.3, 14.9) | 0.008[b] | 203 | 7.8 (6.8, 8.9) |
| Grade 3 | 7 | 1.0 (0.5, 2.2) | 4 | 1.1 (0.4, 2.8) | >0.99 | 8 | 0.3 (0.2, 0.6) |
| Grade 4 | 3 | 0.4 (0.1, 1.3) | 0 | 0 (0, 1.1) | 0.6 | 0 | 0 (0, 0.1) |
| Missing | 0 | 0 (0, 0.6) | 0 | 0 (0, 1.1) | NA | 5 | 0.2 (0.1, 0.4) |

[a] Pyronaridine-artesunate (PA) versus artemether-lumefantrine (AL).

[b] Relative risk 1.6 (95%CI 1.3, 2.2); NC, not applicable.

**Grade 3 Adverse Events**

| Primary system class and preferred term | Integrated safety analysis | | SP-C-021-15 (N=2599) |
|---|---|---|---|
| | PA (N=667) | AL (N=358) | |
| Transaminases increased | 2 (0.3) | 0 | 0 |
| Anaemia | 1 (0.1) | 0 | 2 (0.1) |
| Neutropenia | 1 (0.1) | 0 | 0 |
| Thrombocytopenia | 1 (0.1) | 0 | 0 |
| Multi-organ failure | 1 (0.1) | 0 | 0 |
| Malaria | 1 (0.1) | 0 | 3 (0.1) |
| ALT increased | 1 (0.1) | 1 (0.3) | 0 |
| AST increased | 1 (0.1) | 1 (0.3) | 0 |
| Acarodermatitis | 0 | 1 (0.3) | 0 |
| Vomiting | 0 | 1 (0.3) | 1 (<0.1) |
| Dermatosis | 0 | 1 (0.3) | 0 |
| Toxic epidermial necrolysis | 0 | 1 (0.3) | 0 |
| Asthenia | 0 | 0 | 1 (<0.1) |
| Pyrexia | 0 | 0 | 1 (<0.1) |
| Sepsis | 0 | 0 | 1 (<0.1) |
| Seizure | 0 | 0 | 1 (<0.1) |

Values are n (%). Patients may have had more than one Grade 3 adverse event.

**Grade 4 Adverse Events**

| Primary system class and preferred term | Integrated safety analysis | |
|---|---|---|
| | PA (N=667) | AL (N=358) |
| Drug-induced liver failure | 1 (0.1) | 0 |
| Malaria | 3 (0.4) | 0 |

Values are n (%).
