# Supplementary material for: Safety and efficacy of pyronaridine–artesunate paediatric granules in the treatment of uncomplicated malaria in children: insights from randomized clinical trials and a real-world study
Source: Malar J. 2024 Feb 28;23:61. doi: 10.1186/s12936-024-04885-3 (PMC10902982; doi:10.1186/s12936-024-04885-3)
Supplement: Supplementary file 4 — Additional file 4. Incidence of drug-related adverse events by MedDRA primary system organ class and preferred term in the integrated safety analysis of SP-C-003-05, SP-C-007-07, and WANECAM (SP-C-013-11) comparing PA with AL. Results for the PA real-world study CANTAM (SP-C-021-15) are also shown. [file 12936_2024_4885_MOESM4_ESM.pdf]

Additional file 4. Incidence of drug-related adverse events by MedDRA primary system organ class and preferred term in the integrated safety analysis of SP-C-003-05, SP-C-007-07, and WANECAM (SP-C-013-11) comparing PA with AL. Results for the PA real-world study CANTAM (SP-C-021-15) are also shown.

Primary system class and preferred term	Integrated safety analysis			SP-C-021-15 (N=2599)
	PA (N=667)	AL (N=358)	*P* value[a]	
At least one drug related adverse event	200 (30.0)	134 (37.4)	0.017	218 (8.4)
Blood and lymphatic system disorders	45 (6.7)	34 (9.5)	0.14	4 (0.2)
Anaemia	16 (2.4)	13 (3.6)	0.32	4 (0.2)
Neutropenia	15 (2.2)	8 (2.2)	1.0	0
Monocytosis	8 (1.2)	9 (2.5)	0.13	0
Leukopenia	4 (0.6)	0	0.30	0
Thrombocytopenia	4 (0.6)	2 (0.6)	1.0	0
Splenomegaly	3 (0.4)	0	0.56	0
Eosinophilia	2 (0.3)	1 (0.3)	1.0	0
Leukocytosis	1 (0.1)	0	1.0	0
Lymphopenia	1 (0.1)	0	1.0	0
Basophilia	0	2 (0.6)	0.12	0
Lymphocytosis	0	3 (0.8)	0.042	0
Cardiac disorders	0	1 (0.3)	0.35	0
Arrhythmia	0	1 (0.3)	0.35	0
Ear and labyrinth disorders	0	0	NA	1 (<0.1)
Ear congestion	0	0	NA	1 (<0.1)
Eye disorders	1 (0.1)	0	1.0	0
Conjunctivitis	1 (0.1)	0	1.0	0
Gastrointestinal disorders	42 (6.3)	17 (4.7)	0.33	140 (5.4)
Vomiting	32 (4.8)	11 (3.1)	0.25	109 (4.2)
Abdominal pain	6 (0.9)	4 (1.1)	0.75	7 (0.3)
Diarrhoea	2 (0.3)	3 (0.8)	0.35	21 (0.8)
Aphthous stomatitis	1 (0.1)	1 (0.3)	1.0	0
Constipation	1 (0.1)	0	1.0	1 (<0.1)
Gastritis	1 (0.1)	0	1.0	0
Faeces discoloured	0	0	NA	1 (<0.1)
Haematochezia	0	0	NA	1 (<0.1)
Nausea	0	0	NA	2 (0.1)
Oral disorder	0	0	NA	1 (<0.1)
Tongue ulceration	0	0	NA	1 (<0.1)
General disorders and administration site conditions	4 (0.6)	0	0.30	45 (1.7)
Fatigue	2 (0.3)	0	0.55	3 (0.1)
Chest pain	1 (0.1)	0	1.0	0
Pyrexia	1 (0.1)	0	1.0	25 (1.0)
Swelling	1 (0.1)	0	1.0	0
Asthenia	0	0	NA	12 (0.5)
Influenza like illness	0	0	NA	5 (0.2)
Hepatobiliary disorders	1 (0.1)	0	1.0	0
Drug-induced liver injury	1 (0.1)	0	1.0	0

Hyperbilirubinaemia	1 (0.1)	0	1.0	0
Infections and infestations	27 (4.0)	19 (5.3)	0.35	13 (0.5)
Upper respiratory tract infection	11 (1.6)	4 (1.1)	0.59	0
Bronchitis	7 (1.0)	11 (3.1)	0.02	0
Rhinitis	3 (0.4)	4 (1.1)	0.25	0
Respiratory tract infection	2 (0.3)	0	0.55	0
Tinea capitis	2 (0.3)	0	0.55	0
Gastroenteritis	1 (0.1)	0	1.0	0
Malaria	1 (0.1)	0	1.0	0
Oral herpes	1 (0.1)	0	1.0	0
Pyoderma	1 (0.1)	0	1.0	0
Urinary tract infection	1 (0.1)	2 (0.6)	0.28	1 (<0.1)
Nasopharyngitis	0	1 (0.3)	0.35	3 (0.1)
Oral candidiasis	0	1 (0.3)	0.35	1 (<0.1)
Abscess	0	0	NA	1 (<0.1)
Abscess oral	0	0	NA	1 (<0.1)
Influenza	0	0	NA	2 (0.1)
Pharyngitis	0	0	NA	1 (<0.1)
Pneumonia	0	0	NA	1 (<0.1)
Tonsillitis	0	0	NA	3 (0.1)
Injury, poisoning and procedural complications	1 (0.1)	0	1.0	0
Mouth injury	1 (0.1)	0	1.0	0
Investigations	100 (15.0)	72 (20.1)	0.043	1 (<0.1)
Blood glucose decreased	25 (3.7)	12 (3.4)	0.86	0
Platelet count increased	25 (3.7)	13 (3.6)	1.0	0
Aspartate aminotransferase increased	21 (3.1)	15 (4.2)	0.38	0
Electrocardiogram QT prolonged	20 (3.0)	28 (7.8)	0.0009	0
Blood albumin decreased	15 (2.2)	14 (3.9)	0.17	0
Alanine aminotransferase increased	14 (2.1)	6 (1.7)	0.81	0
Blood potassium increased	14 (2.1)	4 (1.1)	0.32	0
Haemoglobin decreased	13 (1.9)	5 (1.4)	0.62	1 (<0.1)
Haematocrit decreased	12 (1.8)	4 (1.1)	0.60	0
White blood cell count increased	8 (1.2)	1 (0.3)	0.17	0
Platelet count decreased	6 (0.9)	1 (0.3)	0.43	0
Blood creatinine decreased	5 (0.7)	6 (1.7)	0.21	0
Transaminases increased	5 (0.7)	0	0.17	0
Blood creatine phosphokinase increased	2 (0.3)	0	0.55	0
Red blood cell count increased	2 (0.3)	0	0.55	0
Blood alkaline phosphatase increased	1 (0.1)	2 (0.6)	0.28	0
Blood creatinine increased	1 (0.1)	0	1.0	0
Blood glucose increased	1 (0.1)	2 (0.6)	0.28	0
Blood potassium decreased	1 (0.1)	0	1.0	0
Eosinophil count increased	1 (0.1)	0	1.0	0
Red blood cell count decreased	1 (0.1)	1 (0.3)	1.0	0
Blood bilirubin increased	0	2 (0.6)	0.12	0
Blood sodium increased	0	2 (0.6)	0.12	0

Blood urea increased	0	1 (0.3)	0.35	0
Reticulocyte count increased	0	1 (0.3)	0.35	0
Metabolism and nutrition disorders	4 (0.6)	2 (0.6)	1.0	6 (0.2)
Decreased appetite	3 (0.4)	1 (0.3)	1.0	5 (0.2)
Hypercreatininaemia	1 (0.1)	0	1.0	0
Hyperkalaemia	0	1 (0.3)	0.35	0
Dehydration	0	0	NA	1 (<0.1)
Nervous system disorders	5 (0.7)	1 (0.3)	0.67	8 (0.3)
Headache	4 (0.6)	1 (0.3)	0.66	6 (0.2)
Somnolence	1 (0.1)	0	1.0	1 (<0.1)
Dizziness	0	0	NA	2 (0.1)
Renal and urinary disorders	2 (0.3)	0	0.55	0
Ketonuria	1 (0.1)	0	1.0	0
Proteinuria	1 (0.1)	0	1.0	0
Respiratory, thoracic and mediastinal disorders	4 (0.6)	3 (0.8)	0.70	9 (0.3)
Cough	2 (0.3)	1 (0.3)	1.0	5 (0.2)
Asthma	1 (0.1)	0	1.0	0
Epistaxis	1 (0.1)	1 (0.3)	1.0	1 (<0.1)
Rhinorrhoea	0	1 (0.3)	0.35	1 (<0.1)
Dyspnoea	0	0	NA	1 (<0.1)
Oropharygeal pain	0	0	NA	1 (<0.1)
Skin and subcutaneous tissue disorders	3 (0.4)	4 (1.1)	0.25	17 (0.7)
Rash	2 (0.3)	0	0.55	3 (0.1)
Blister	1 (0.1)	0	1.0	0
Dermatosis	0	1 (0.3)	0.35	0
Pruritus	0	1 (0.3)	0.35	5 (0.2)
Rash pruritic	0	1 (0.3)	0.35	1 (<0.1)
Toxic epidermal necrolysis	0	1 (0.3)	0.35	0
Hyperidrosis	0	0	NA	3 (0.1)
Dermatitis	0	0	NA	1 (<0.1)
Miliaria	0	0	NA	1 (<0.1)
Pruritus generalised	0	0	NA	1 (<0.1)
Skin swelling	0	0	NA	1 (<0.1)
Swelling face	0	0	NA	1 (<0.1)
Urticaria	0	0	NA	1 (<0.1)

[a] Pyronaridine-artesunate (PA) versus artemether-lumefantrine (AL).
NA, not applicable.
Vales are n (%).
