# Supplementary material for: Safety and efficacy of pyronaridine–artesunate paediatric granules in the treatment of uncomplicated malaria in children: insights from randomized clinical trials and a real-world study
Source: Malar J. 2024 Feb 28;23:61. doi: 10.1186/s12936-024-04885-3 (PMC10902982; doi:10.1186/s12936-024-04885-3)
Supplement: Supplementary file 5 — Additional file 5. Changes from baseline for haematology and biochemistry in the integrated safety analysis of SP-C-003-05, SP-C-007-07, and WANECAM (SP-C-013-11). [file 12936_2024_4885_MOESM5_ESM.pdf]

Additional file 5. Changes from baseline for haematology and biochemistry in the integrated safety analysis of SP-C-003-05, SP-C-007-07, and WANECAM (SP-C-013-11).

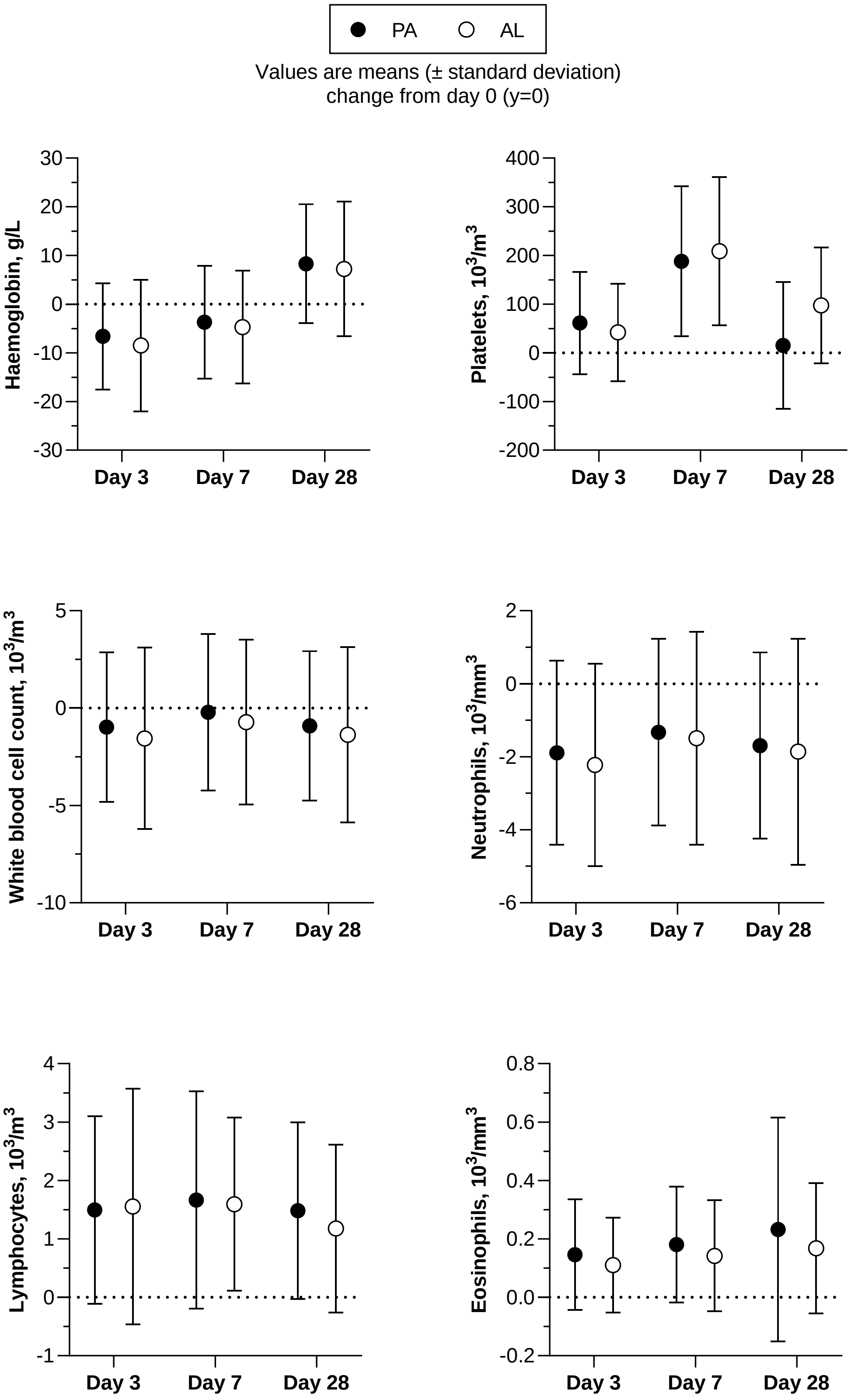

.

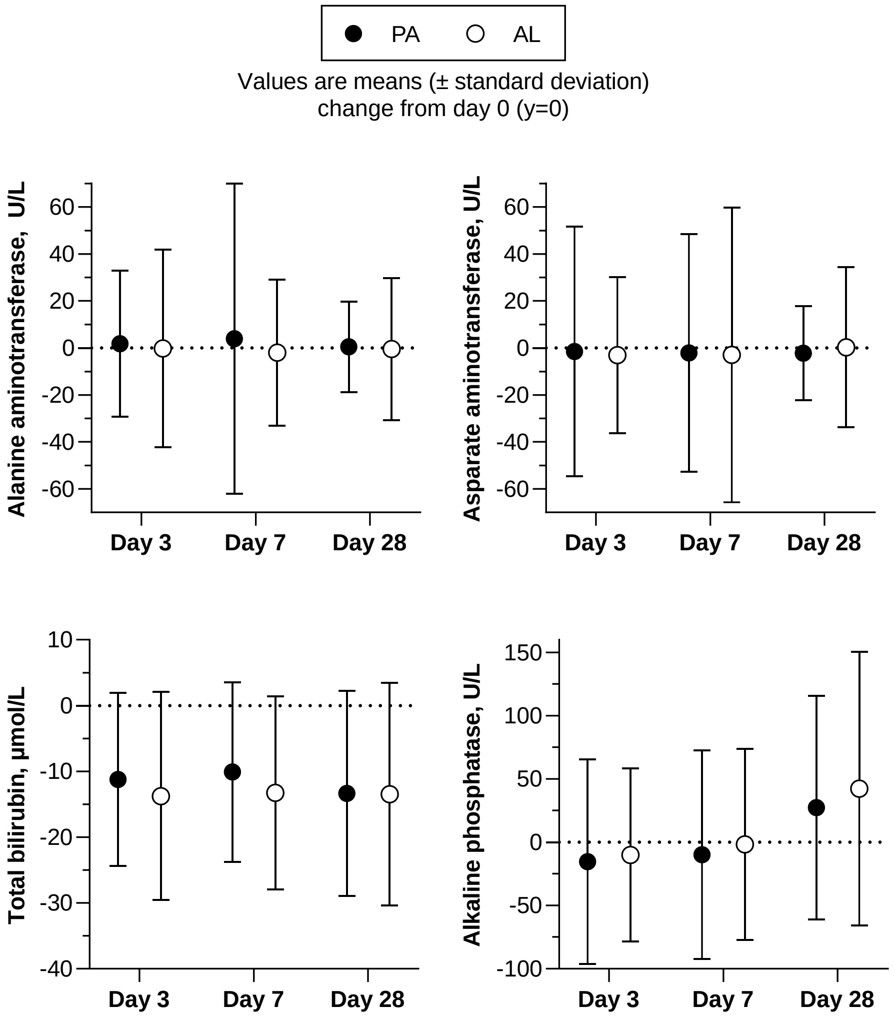
PA
AL
Values are means (± standard deviation)
change from day 0 (y=0)
Alanine aminotransferase, U/L
Asparate aminotransferase, U/L
Total bilirubin, µmol/L
Alkaline phosphatase, U/L
60
40
20
0
-20
-40
-60
10
-10
-30
-40
150
100
50
-50
-100
Day 3
Day 7
Day 28
