# Supplementary material for: Safety and efficacy of pyronaridine–artesunate paediatric granules in the treatment of uncomplicated malaria in children: insights from randomized clinical trials and a real-world study
Source: Malar J. 2024 Feb 28;23:61. doi: 10.1186/s12936-024-04885-3 (PMC10902982; doi:10.1186/s12936-024-04885-3)
Supplement: Supplementary file 6 — Additional file 6. Frequency of baseline elevations in alanine aminotransferase and aspartate aminotransferase in CANTAM (SP-C-021-15). [file 12936_2024_4885_MOESM6_ESM.pdf]

Additional file 6. Frequency of baseline elevations in alanine aminotransferase and aspartate aminotransferase in CANTAM (SP-C-021-15).

Enzyme	Value relative to xULN	Evaluable patients (N=2586)
Alanine aminotransferase	≤1.5	2547 (98.5)
	>1.5 to ≤3	21 (0.8)
	>3 to ≤5	5 (0.2)
	>5 to ≤10	7 (0.3)
	>10	6 (0.2)
Aspartate aminotransferase	≤1.5	2410 (93.2)
	>1.5 to ≤3	138 (5.3)
	>3 to ≤5	24 (0.9)
	>5 to ≤10	8 (0.3)
	>10	5 (0.2)

Values are n (%). ULN, upper limit of normal.
