# Supplementary figures and images for: Safety and efficacy of pyronaridine–artesunate paediatric granules in the treatment of uncomplicated malaria in children: insights from randomized clinical trials and a real-world study
Source: Malar J. 2024 Feb 28;23:61. doi: 10.1186/s12936-024-04885-3 (PMC10902982; doi:10.1186/s12936-024-04885-3)

Additional file 7. WANECAM (SP-C-013-11) patient disposition.


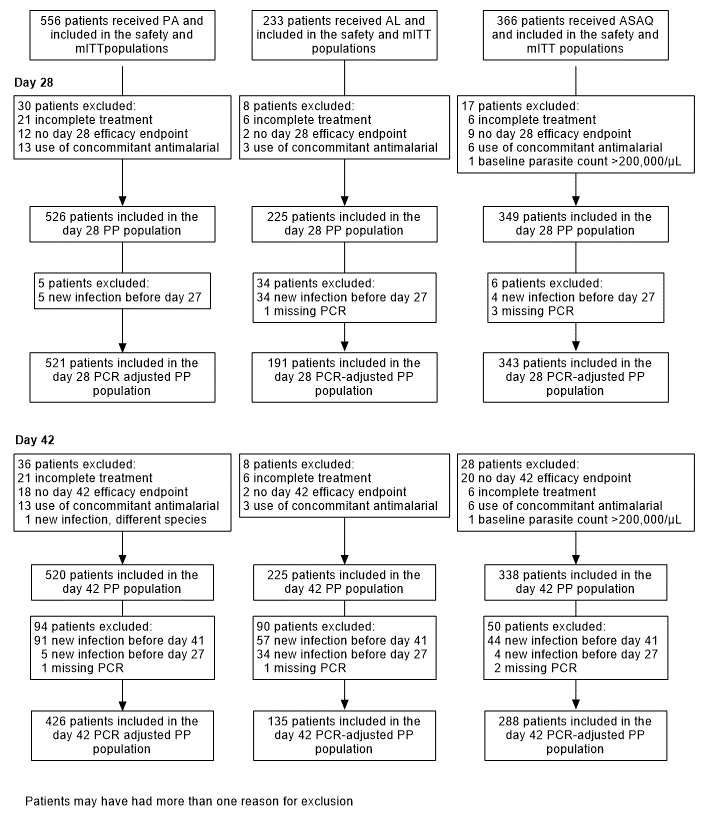

Supplement: Supplementary file 7 — Additional file 7. WANECAM (SP-C-013-11) patient disposition. [file 12936_2024_4885_MOESM7_ESM.docx]
