# Supplementary material for: Safety and efficacy of pyronaridine–artesunate paediatric granules in the treatment of uncomplicated malaria in children: insights from randomized clinical trials and a real-world study
Source: Malar J. 2024 Feb 28;23:61. doi: 10.1186/s12936-024-04885-3 (PMC10902982; doi:10.1186/s12936-024-04885-3)
Supplement: Supplementary file 8 — Additional file 8. WANECAM (SP-C-013-11) baseline characteristics (day 28 per protocol population). [file 12936_2024_4885_MOESM8_ESM.docx]

Additional file 8. WANECAM (SP-C-013-11) baseline characteristics (day 28 per protocol population).

| **Characteristic** | **PA (N=536)** | **AL (N=227)** | **ASAQ (N=352)** |
| --- | --- | --- | --- |
| Male sex, n (%) | 256 (47.8) | 118 (52.0) | 191 (54.3) |
| Female sex, n (%) | 280 (52.2) | 109 (48.0) | 161 (45.7) |
| Mean age (SD) [range], years | 4.1 (1.8) [0–10] | 4.4 (1.8) [0–11] | 3.9 (1.9) [0–11] |
| Age category, n (%) |  |  |  |
| ≤6 months | 0 | 0 | 0 |
| >6 months to <1 year | 9 (1.7) | 2 (0.9) | 11 (3.1) |
| ≥1 to <3 years | 106 (19.8) | 30 (13.2) | 70 (19.9) |
| ≥3 to < 6 years | 308 (57.5) | 140 (61.7) | 206 (58.5) |
| ≥6 years | 113 (21.1) | 55 (24.2) | 65 (18.5) |
| Mean body weight (SD) [range], kg | 14.6 (3.1)  [6.7–19.9] | 15.0 (2.8)  [7.8–19.8] | 14.2 (3.2)  [5.9–19.9] |
| Body weight category, n (%) |  |  |  |
| <8 kg | 6 (1.1) | 1 (0.4) | 8 (2.3) |
| ≥8 to <15 kg | 261 (48.7) | 103 (45.4) | 186 (52.8) |
| ≥15 to <20 kg | 269 (50.2) | 123 (54.2) | 158 (44.9) |
| Episode 1 *Plasmodium* spp., n (%) |  |  |  |
| *P. falciparum* asexual | 529 (98.7) | 227 (100) | 346 (98.3) |
| *P. falciparum* gametocytes | 14 (2.6) | 6 (2.6) | 15 (4.3) |
| *P. ovale* asexual | 1 (0.2) | 1 (0.4) | 2 (0.6) |
| *P. ovale* gametocytes | 1 (0.2) | 0 | 0 |
| *P. malariae* asexual | 15 (2.8) | 5 (2.2) | 9 (2.6) |
| *P. malariae* gametocytes | 2 (0.4) | 0 | 1 (0.3) |
| Episode 1 geometric mean *P. falciparum* asexual parasitemia [range], /µL | 14,659  [40–198,960] | 21,493  [16–198,960] | 11,046  [32–317,800] |

Values are n (%).

AL, artemether-lumefantrine; ASAQ, artesunate-amodiaqune; PA, pyronaridine-artesunate.
